# Integrated Analysis of Metabolome and Transcriptome Reveals Insights for Cold Tolerance in Rapeseed (*Brassica napus* L.)

**DOI:** 10.3389/fpls.2021.721681

**Published:** 2021-10-08

**Authors:** Ali Raza, Wei Su, Muhammad Azhar Hussain, Sundas Saher Mehmood, Xuekun Zhang, Yong Cheng, Xiling Zou, Yan Lv

**Affiliations:** ^1^Key Laboratory of Biology and Genetic Improvement of Oil Crops, Ministry of Agriculture, Oil Crops Research Institute, Chinese Academy of Agricultural Sciences (CAAS), Wuhan, China; ^2^College of Agriculture, Engineering Research Center of Ecology and Agricultural Use of Wetland of Ministry of Education, Yangtze University, Jingzhou, China

**Keywords:** abiotic stress, carbohydrate metabolism, metabolites, phenylalanine metabolism, omics

## Abstract

Rapeseed (*Brassica napus* L.) is an important oilseed crop in the world. Its productivity is significantly influenced by numerous abiotic stresses, including cold stress (CS). Consequently, enhancement in CS tolerance is becoming an important area for agricultural investigation and crop improvement. Therefore, the current study aimed to identify the stress-responsive genes, metabolites, and metabolic pathways based on a combined transcriptome and metabolome analysis to understand the CS responses and tolerance mechanisms in the cold-tolerant (C18) and cold-sensitive (C6) rapeseed varieties. Based on the metabolome analysis, 31 differentially accumulated metabolites (DAMs) were identified between different comparisons of both varieties at the same time points. From the transcriptome analysis, 2,845, 3,358, and 2,819 differentially expressed genes (DEGs) were detected from the comparison of C6-0 vs. C18-0, C6-1 vs. C18-1, and C6-7 vs. C18-7. By combining the transcriptome and metabolome data sets, we found that numerous DAMs were strongly correlated with several differentially expressed genes (DEGs). A functional enrichment analysis of the DAMs and the correlated DEGs specified that most DEGs and DAMs were mainly enriched in diverse carbohydrates and amino acid metabolisms. Among them, starch and sucrose metabolism and phenylalanine metabolism were significantly enriched and played a vital role in the CS adaption of rapeseed. Six candidate genes were selected from the two pathways for controlling the adaption to low temperature. In a further validation, the T-DNA insertion mutants of their *Arabidopsis* homologous, including *4cl3, cel5, fruct4, ugp1, axs1*, and *bam2/9*, were characterized and six lines differed significantly in levels of freezing tolerance. The outcome of the current study provided new prospects for the understanding of the molecular basis of CS responses and tolerance mechanisms in rapeseed and present a set of candidate genes for use in improving CS adaptability in the same plant.

## Introduction

Rapeseed (*Brassica napus* L.) is an essential oilseed crop. Due to the high oil content in its seeds, it is considered to account for one-third of the total edible oils throughout the world, which helps in the mass production of animal feed and vegetable oil (Jiang et al., 2014). Rapeseed is sensitive to several abiotic stresses, such as drought, salinity, flood, and cold, severely reducing the growth and production of the crop, thus resulting in agricultural economic loss and production risks (Raza, 2020a). Among the different abiotic stresses, cold stress (CS) is one of the critical abiotic stresses in China, as it limits crop species, growth, yield, and quality (Yang et al., 2015). Cold tolerance is an essential trait for crops to withstand low temperatures, especially for overwintering crops (Sun et al., 2010; He et al., 2021). In general, CS thermodynamically decreases the kinetics of the various physiological and metabolic processes occurring in plants (Ruelland et al., 2009), which, accordingly, cause cell membrane rigorousness, protein complex uncertainty, and photosynthesis weakness (Ruelland et al., 2009). Subsequently, CS seriously reduces the rate and uniformity of germination, hampers seedling vigor, and delays plant development, resulting in serious crop yield losses (He et al., 2021; Raza et al., 2021b). For instance, severe winter freezing temperatures affected 77.8% of the rapeseed growing area and caused significant production and yield loss in China (Zhang et al., 2008). Furthermore, the late sowing of winter rapeseed reduced the rapeseed yield, and early flowering was also significantly affected by CS. Moreover, intracellular ice crystals are formed due to CS, distorting the sensitive plant organs (Lardon and Triboi-Blondel, 1995; Raza, 2020a; Shafighi et al., 2020). New seedlings, flowering, and seed formation are also mainly affected by CS (Lardon and Triboi-Blondel, 1995; Shafighi et al., 2020). In recent studies, it was found that CS significantly reduced survival rate by impairing different physio-biochemical (reduced antioxidant enzyme activities, osmolyte content, and phytohormone levels) and molecular (reduced transcript levels of CS-responsive marker and antioxidant genes) mechanisms (Yan et al., 2019; He et al., 2021). Furthermore, CS also greatly affects the photosynthetic rate, stomatal conductance, transpiration rate, and chlorophyll fluorescence parameters in rapeseed (Mehmood et al., 2021). Therefore, it is urgent to develop CS-tolerant rapeseed varieties to ensure yield under such unfavorable conditions.

Several plants showed improved chilling tolerance after an experience with chilling stress in a mechanism called cold acclimation (Ruelland et al., 2009) which includes a collection of physiological and biochemical adjustments. These changed responses involve deviations in gene expression trends *via* the ICE-CBF-COR regulatory pathway (Shi et al., 2018). The C-repeat binding factors (CBF) transcription factors, also recognized as dehydration responsive element binding factor 1 (DREB1) proteins belonging to the AP2/ERF domain family, are meaningfully induced under CS (Chinnusamy et al., 2010; Akhtar et al., 2012; Shi et al., 2018). Other than regulatory networks, plants have modulated their biochemical ways in response to CS. For instance, carbohydrates, lipids, and amino acids have been extensively described to be elaborated in multiple abiotic stress responses, not only for protein amalgamation procedure, but also as pioneers of further key metabolites under stress conditions (Zhao et al., 2007; Bhandari et al., 2016; Zhang et al., 2017). However, more CS-related metabolites need to be comprehensively explained in future research.

Nowadays, high-throughput omics approaches, including genomics, transcriptomics, proteomics, and metabolomics, have been widely used by plant researchers to study different abiotic stresses and deepen their understanding of diverse biological pathways (Raza et al., 2021a,b). Recently, two groups used the integrative comparative analysis of metabolite and transcript profiles to dissect the CS-tolerance mechanisms in tomatoes (*Solanum lycopersicum* L.) (Zhang W.-F. et al., 2019) and wheat (*Triticum aestivum* L.) (Zhao et al., 2019) under CS conditions. These studies identify different amino acids, sugars, organic acids, and lipid-related compounds and metabolic pathways under CS conditions. In another study, the complex molecular mechanisms of metabolites and candidate genes involved in the salt response of sesame have been explored (Zhang Y. et al., 2019). Overall, these integrated studies have expanded our knowledge and understanding of the complex regulatory mechanisms that are used to respond to stressful environments. To date, significant progress has been made toward the conjoint analysis of metabolome and transcriptome in response to different abiotic stresses in *Arabidopsis*, Ethiopian mustard (*B. carinata*), oat (*Avena sativa*), sorghum (*Sorghum bicolor*), and Chinese woad (*Isatis indigotica* Fort.) (Hirai et al., 2004; Li et al., 2009, 2019; Zhou et al., 2015; Wang et al., 2018a). However, studies on the molecular mechanisms of CS response in *Brassica* crops, mainly in rapeseed under CS conditions, have made few advances. Comparative approaches, therefore, should be used in the research of complex molecular mechanisms responses to CS in rapeseed. Nevertheless, the integrated omics analysis (transcriptome and metabolome) of rapeseed under CS could be considered a fascinating approach to revealing key CS-responsive mechanisms at the molecular and metabolic levels.

In the present study, the transcriptome and metabolome profiles of the seedlings of a cold-tolerant and a sensitive rapeseed variety were performed in the early phase of CS. Conjoint analysis of metabolome and transcriptome permitted us to identify putative metabolites, genes, metabolic pathways, and their interactions in response to CS. To the of our best knowledge, this is the first time that a correlation analysis between identified genes and metabolites from the combined data set under CS, mainly in rapeseed, was carried out. This study provides novel insights into the mechanisms underlying rapeseed CS adaptation, thus facilitating the development of CS-tolerant rapeseed varieties.

## Materials and Methods

### Plant Material, Stress Treatments, and Evaluation of Some Physiological Indexes

Two rapeseed varieties, C18 (cold tolerant; CT-C18) and C6 (cold-sensitive; CS-C6), were used to analyze the defense mechanisms against CS. The seeds of both varieties were obtained from the Oil Crops Research Institute, Chinese Academy of Agricultural Science (CAAS), Wuhan, China. The rapeseed seeds were sterilized with distilled sterilized water for 10 min. Then, the seeds were germinated on two wet filter papers in a Petri dish under normal conditions for 8 days. After that the uniform seedlings were transferred to small pots containing soil in a growth chamber with a 16/8-h light/dark cycle at 25°C. Twenty-one-day-old seedlings were then treated at 8/4°C day/night for 7 days. Samples were collected at 0 (control/CK), 1, and 7 days after the treatment. Our findings were parallel with a recent report (Tian et al., 2017), which found that rapeseed CK plants did not show any significant difference in morphological attributes when harvested under different temperatures and light intensities. Thus, only one CK for the entire analysis was kept. Three biological replicates were harvested for transcriptomic and metabolomic analysis, which was performed in different comparisons (Supplementary Table 1). All the samples were immediately frozen in liquid nitrogen and stored at −80°C for further use.

The above-mentioned harvested samples were used for evaluating some physiological indices in both varieties. The activities of superoxide dismutase (SOD, EC 1.15.1.1; cat no. G0101F), and peroxidase (POD, EC 1.11.1.7; cat no. G0107F), the contents of malondialdehyde (MDA, cat no. G0109F), soluble sugar (cat no. G0501W), soluble protein (cat no. G0418W), and proline (cat no. G0111W) were measured using the kits provided by Suzhou Grace Biotechnology Co., Ltd. (Suzhou, Jiangsu, China). All the parameters were measured using three technical replications and a spectrophotometer microplate reader (Epoch, BioTek, Instruments, Inc., Winooski, VT, USA).

### Total RNA Isolation and Transcriptome Sequencing

Total RNA was isolated from each sample using the HiPure Plant RNA Mini Kit (Magen R4151-03, Zhengzhou, China) according to the instructions of the manufacturer. The RNA concentration was measured using the NanoDrop 2000 (Thermo Scientific, USA). The RNA integrity was assessed using the RNA Nano 6000 Assay Kit of the Agilent Bioanalyzer 2100 Bioanalyzer (Agilent Technologies, CA, USA). For RNA-seq/transcriptome and metabolome analysis, the three biological samples were sent to BioMarker Technologies (Beijing, China). Lastly, RNA-seq was conducted on the Illumina HiSeq high throughput sequencing platform (HiSeq 2000, SanDiego, CA, USA).

### Transcriptome Assembly, Annotation, and Differential Expression Analysis of the Genes

Raw data (raw reads) of the fastq format were first processed through in-house perl scripts. In this step, clean data (clean reads) were attained by removing reads comprising adapter, reads containing ploy-N, and low-quality reads from raw data. Simultaneously, Q20, Q30, GC-content, and sequence duplication levels of the clean data were calculated. All the downstream analyses were based on clean data with high quality. The adaptor sequences and low-quality sequence reads were removed from the data sets. The clean reads were then mapped to the reference genome sequence “Darmor-bzh” (http://www.genoscope.cns.fr/brassicanapus/) (Chalhoub et al., 2014). Only reads with a perfect match or one mismatch were further analyzed and annotated based on the reference genome using the Hisat2 V2.1.0 tool.

Gene function was annotated based on the following databases: Pfam (Protein family) (https://pfam.xfam.org/); Swiss- Prot (A manually annotated and reviewed protein sequence database) (https://www.uniprot.org/); KO [Kyoto Encyclopedia of Genes and Genomes (KEGG) Ortholog database] (https://www.genome.jp/kegg/ko.html); GO (Gene Ontology) (http://www.geneontology.org). The quantification of the gene expression levels was projected by fragments per kilobase of transcript (FPKM) per million fragments mapped.

The differential expression analysis of two conditions or groups was completed using the R package DEseq. The resulting *p*-values were adjusted using the approach of Benjamini and Hochberg for controlling the false discovery rate (FDR). Genes with log_2_FC > 4, adjusted *p* < 0.001, and FDR < 0.001 were assigned as differentially expressed genes (DEGs).

### Metabolite Extraction

The above-mentioned samples were also considered for metabolome analysis. Briefly, 50 mg of the sample were taken and placed in an EP tube, then added with 1,000 μl extraction solvent containing an internal target (*V* methanol: *V* acetonitrile: *V* water = 2:2:1, containing internal standard 2 μg ml^−1^). They were then homogenized in a ball mill for 4 min at 45 Hz, then ultrasound treated for 5 min (incubated in ice water). After homogenizing 3 times, they were incubated for 1 h at −20°C to precipitate proteins. They were then centrifuged at 12,000 rpm for 15 min at 4°C. After which, the supernatant (500 μl) was transferred fresh into the EP tubes. The extracts were dried in a vacuum concentrator without heating and added with 100 μl extraction solvent (*V* acetonitrile: *V* water = 1:1) reconstitution. The samples were then vortexed for 30 s, sonicated for 10 min (4°C water bath), and centrifuged for 15 min at 12,000 rpm, 4°C. Afterward, the supernatants (60 μl) were transferred into a fresh 2-ml LC/MS glass vial. Lastly, 10 μl from each sample were then taken and pooled as QC samples and 60 μl of supernatant were taken for an ultra-high-performance liquid tandem chromatography-quadrupole time of flight-mass spectrometry (UHPLC-QTOF-MS) analysis.

### Metabolite Detection

The LC-MS/MS analyses were performed using a UHPLC system (1290, Agilent Technologies) with a UPLC BEH Amide column (1.7 μm 2.1 ^*^ 100 mm, Waters) coupled to TripleTOF 6600 (Q-TOF, AB Sciex). The mobile phase consisted of 25 mM NH_4_Ac and 25 mM NH_4_OH in water (pH = 9.75) (A) and acetonitrile (B) was carried with elution gradient as follows: 0 min, 95% B; 0.5 min, 95% B; 7 min, 65% B; 8 min, 40% B; 9 min, 40% B; 9.1 min, 95% B; 12 min, 95% B, delivered at 0.5 ml min^−1^. The injection volume was pos:1.5 μl, neg: 1 μl. The Triple TOF mass spectrometer was used for its ability to acquire MS/MS spectra on an information-dependent basis (IDA) during an LC/MS experiment. In this mode, the acquisition software (Analyst TF 1.7, AB Sciex) continuously evaluates the full scan survey MS data as it collects and triggers the acquisition of MS/MS spectra depending on preselected criteria. In each cycle, 12 precursor ions with intensities <100 were chosen for fragmentation at a collision energy (CE) of 30 V (15 MS/MS events with product ion accumulation time of 50 ms each). The ESI source conditions were set as follows: ion source gas 1 as 60 psi, ion source gas 2 as 60 psi, curtain gas as 35 psi, source temperature at 650°C, and Ion Spray Voltage Floating (ISVF) 5,000 or −4,000 V in positive or negative modes, respectively.

### Bioinformatics Analysis of Combined Data

The metabolome data were normalized before analysis through the normalizing of the total peak area by dividing each metabolite in the sample by the total peak area of the sample. PCA, cluster heatmap analysis, correlation analysis between samples, and Orthogonal Partial Least Squares-Discriminant Analysis (OPLS-DA) were carried out for classification and discrimination between the samples using R package ropls (R 3.3.2.; http://bioconductor.org/packages/release/bioc/html/ropls.html) (Thevenot, 2016). We combined the multivariate statistical analysis of the VIP value of OPLS-DA and the univariate statistical analysis of the *t*-test *P*-value to screen differentially accumulated metabolites (DAMs) among different comparison groups. The screening criteria FC > 1.5, *P* < 0.05, and VIP > 1 were considered to determine the DAMs between samples.

Gene ontology (GO) enrichment analysis of the differentially expressed genes (DEGs) was executed by the GOseq R package (https://bioconductor.org/packages/release/bioc/html/goseq.html) based on the Wallenius non-central hyper-geometric distribution, which can adjust for gene length bias in DEGs (Anders and Huber, 2010). The KEGG pathway enrichment analysis of the DEGs and DAMs was carried out using the KOBAS software (Mao et al., 2005). The heatmap was created using the TBtool V0.66839 software (Chen et al., 2020).

### Co-expression Network Visualization

The gene expression data (log_2_FC) of the targeted metabolites were selected and imported to Cytoscape (Version 3.5.1. https://cytoscape.org/) for differential metabolites and gene correlation network visualization (Shannon et al., 2003). Firstly, log_2_ conversion was performed on the data uniformly before analysis. For the joint analysis between metabolome and transcriptome, the Pearson Correlation Coefficient (PCC) and the corresponding *P*-value were used for screening, and the screening criteria were set at PCC > 0.8.

### MapMan Pathway Visualization and Enrichment Analysis

The MapMan pathway annotator (version 3.6.0 RC1, https://mapman.gabipd.org/mapman/) was used to display the graphical overview of metabolism pathways (Thimm et al., 2004). Release Genome of *Brassica napus* annotation v5 was used as the mapping reference data to group and display metabolism pathways. *B. napus* gene IDs and their log_2_FC values were imported to MapMan as an experimental data set. The Wilcoxon rank-sum test with Benjamini–Hochberg corrected was used to analyze which bins/pathways were differentially enriched between two accessions.

### qRT-PCR Analysis for DEGs

Total RNA was extracted using the RNAprep Pure Plant Kit (Tiangen) according to the instructions of the manufacturer. The first-strand cDNA was reverse transcribed from 1 μg of the total RNA using the First Strand cDNA Synthesis Kit (Thermo Fisher Scientific). The quantitative real-time PCR (qRT-PCR) was carried out as described previously (Wang et al., 2014). The qRT-PCR reaction was carried out using the StepOnePlusReal-Time PCR System (Applied Biosystems, US) on the *Power*SYBR^®^ Green PCR Master Mix (Applied Biosystems, US). The total volume for each reaction was 10 μl, comprising 0.4 μl specific primers, 1 μl cDNA, 5 μl SYBR mixture, and 3.6 μl double-distilled H_2_O. The cycling program were as follows: 10 min at 95°C, followed by denaturation at 95°C for 15 s, annealing, and elongation at 60°C for 1 min (40 cycles). The *BnActin* gene was used as an endo control. The relative expression was calculated using the 2^−ΔΔCt^ method. The primer used for randomly selected genes is listed in Supplementary Table 2.

### Cold-Induced Alteration of Some Metabolite

To evaluate whether CS reconfigures the metabolite contents in leaves, we analyzed L-Kynurenine, L-Tyrosine, N-Acetyl-L-Phe, salicylic acid, and succinic acid contents. The concentrations of these compounds were calculated using the calibration curves generated from corresponding standard solutions, and the detailed protocol is described in Supplementary File 1.

### Functional Validation of Candidate Genes

For the functional validation of candidate genes, the rapeseed gene IDs were used to search the *Arabidopsis* homolog in the rapeseed genome database (Genoscope; https://www.genoscope.cns.fr/brassicanapus/). The resultant *Arabidopsis* genes were used to search the sequence similarities and annotation in the TAIR database using blastP. Based on the annotation and sequence results, the most suitable candidates were selected for functional validation and had their mutation status checked in the SIGnal database (T-DNA Express: *Arabidopsis* Gene Mapping Tool; http://signal.salk.edu/cgi-bin/tdnaexpress). After PCR screening, only homozygous lines were chosen for further analysis. The sequence alignment between respective rapeseed and *Arabidopsis* T-DNA lines was performed using the MegAlign Pro software (https://www.dnastar.com/software/lasergene/megalign-pro/). Furthermore, *Arabidopsis* homozygous mutants *4cl3* (SALK 014297C), *cel5* (SALK 079921C), *fruct4* (SALK 011312C), *ugp1* (SALK 100183C), *axs1* (SALK 000016C), and *bam2/9* (SALK 020838C) were obtained from the Arashare database, China (https://www.arashare.cn/index/), which were genotyped *via* PCR sequencing to check the homozygous status. Moreover, the relative expression of T-DNA mutants was checked in T-DNA lines and wild-type (WT) plants to confirm their knockout status. The primer used for PCR and qRT-PCR analysis are listed in Supplementary Table 2. The *Arabidopsis* ecotype Columbia-0 was used as the WT. All *Arabidopsis* seeds were germinated and grown for 4 weeks in controlled greenhouse conditions under short-day conditions (10 h of light) at a constant temperature of 22°C. The 4-week-old seedlings of all mutants and the WT were exposed to freezing stress (−5°C for 8 h) in a growth chamber followed by recovery at 25°C for 2 days. The phenotype and survival rate of the leaves were observed, and samples were harvested before and after exposure to freezing stress. The survival rate was measured using the following equation:


$$\begin{array}{l} \text{Survival rate }\left(\%\right)=\frac{\text{Number of survived plants}}{\text{Number of originally planted plants}}\text{ x 100} \end{array}$$

After being exposed to freezing stress, most WT plants survived and could continuously grow under normal conditions after recovery, while the mutant plants wilted severely under freezing treatment and even died after recovery. For each mutant, 12 pots (4 plants pot^−1^) were assessed, and the experiment was completed with three replications. The leaves were harvested before stress (CK), after 8 h of freezing stress, and after 2 days of recovery to measure the osmoprotectant substances [soluble sugar (G0501W), soluble protein (G0418W), and proline (G0111W)] using the kits provided by Suzhou Grace Biotechnology Co., Ltd. (Suzhou, Jiangsu, China). All the parameters were measured using three biological replications and a spectrophotometer microplate reader (Epoch, BioTek, Instruments, Inc., Winooski, VT, USA).

### Statistical Analysis

The statistical analysis was performed using GraphPad Prism 9 (https://www.graphpad.com/) (Swift 1997). The experiments were performed with three biological replicates, and plant materials from three seedlings were pooled for each biological replicate. The statistical significance was determined through a one- or two-way ANOVA and Tukey's test. Error bars represent SD (n = 3). The difference was statistically significant as ^****^P < 0.0001, ^***^P ≤ 0.001, ^**^P ≤ 0.01, and ^*^P ≤0.05.

## Results

### Physiological Responses and Metabolic Profiling of C6 and C18 Varieties Under CS

**Figure 1:**
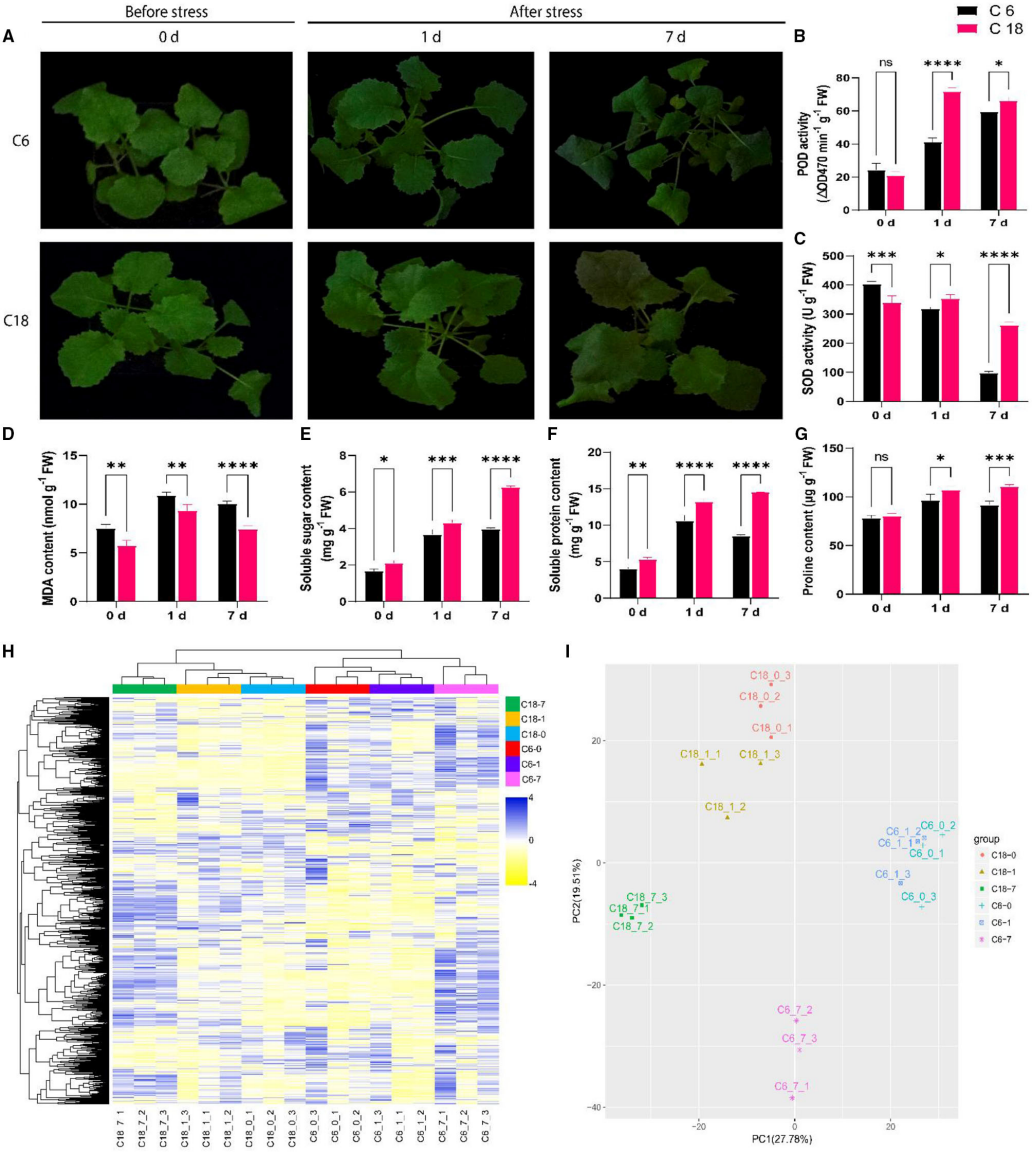
Physiological and metabolic profiling of cold-tolerant (C18) and cold-sensitive (C6) rapeseed varieties responding to cold stress (4°C) at 0 days (CK), 1 day after stress, and 7 days after stress. **(A)** Phenotypic evaluation of C18 and C6; **(B–G)** Physiological responses including **(B)** peroxidase (POD) activity, **(C)** superoxide dismutase (SOD) activity, **(D)** malondialdehyde (MDA) content, **(E)** soluble sugar content, **(F)** soluble protein content, and **(G)** proline content. Error bars represent SD (*n* = 3). The statistical significance was determined *via* a two-way ANOVA and Dunnett's multiple comparisons test with ^****^P < 0.0001, ^****^p ≤ 0.001, ^**^p ≤ 0.01, ^*^p ≤ 0.05, and ns mean non-significant. **(H)** The unsupervised hierarchical cluster analysis for untargeted metabolomic profiles. The dendrogram was built based on the fold change (*P* < 0.05, VIP > 1 and log_2_FoldChange > 1.5) heatmap. The rows in the heatmap indicate metabolites, and the columns represent groups/samples. The colors of the heatmap cells represent the scaled expression level of metabolites among the different groups. The color gradient, ranging from blue to yellow through white, indicates the low, high, and middle values of metabolite expression. The colors of the different groups represent the biological replicates at three different time points for two genotypes, while 0 (CK), 1, and 7 days represent the sample collection time points after the treatment. **(I)** Principal component analysis (PCA) based on metabolomic data. The X-axis represents the first principle component (PC1), and the Y-axis represents the second principal component (PC2).

To examine the variations of rapeseed during CS, two rapeseed varieties were selected. According to previous studies, zhongshuang 6 (C6) was a common transgenic receptor that exhibited susceptibility to CS, whereas C18 was an excellent breeding line that exhibited cold tolerance (Yan et al., 2019). The phenotypic result showed that C6 displayed severe wilting and chlorosis at 7 days of CS compared to C18 (Figure 1A). At physiological levels, CT-C18 performed better than CS-C6 (Figures 1B–G). For instance, the activities of antioxidant enzymes such as SOD and POD were found to be higher in CT-C18 under CS conditions, mainly at 1 and 7 days (Figures 1B,C). The MDA is interpreted as an end product of lipid peroxidation, and regulation of MDA content indicates the oxidative stress levels in plants (Gaweł et al., 2004). Thus, the MDA content was considerably higher in CS-C6 compared to CT-C18 (Figure 1D), indicating that CT-C18 had a better capacity to cope with oxidative stress than CS-C6. Moreover, osmoprotectant content, including soluble sugar, soluble protein, and proline contents, was significantly higher in CT-C18 under CS conditions (Figures 1E–G). These findings showed that CT-C18 is a more cold-tolerant variety than CS-C6 and could mitigate the adverse effects of CS by increasing the activities of antioxidant defense enzymes and content of osmoprotectants and reducing oxidative stress (Yan et al., 2019).

To construct a systematical profile of metabolic changes that occur in response to CS, an untargeted metabolome analysis was performed between cold-sensitive (CS-C6) and cold-tolerant (CT-C18) varieties. In total, 3,368 metabolites were detected; out of these, 626 were known metabolites (Supplementary Table 3, Supplementary Figure 1). These known metabolites belonged to 81 classes, with majority of these metabolites belonging to carboxylic acids and derivatives, organooxygen compounds, fatty acyls, benzene and substituted derivatives, organonitrogen compounds, prenol lipids, purine nucleosides, etc. (Supplementary Table 3, Supplementary Figure 1). To get a better view of the metabolic profiles in response to CS, the unsupervised hierarchical cluster was built, indicating that metabolic data from the early stage of CS (0–1 day) were clearly separated from the late-stage stress (7 days) (Figure 1H). Similarly, the PCA explained 47.29% of the total variance (27.78 and 19.51% for PC1 and PC2, respectively). The PCA showed the precise isolation between the different time points by PC1. The isolation of genotypes from biological replications can be distinguished by PC2 (Figure 1I), which showed a good correlation between replications (Iezzoni and Pritts, 1991; Ringnér, 2008). These results indicated that differential metabolic response between both varieties could be the basis of their different tolerances to CS.

### DAMs Involved in Cold Response Between Two Rapeseed Varieties

**Figure 2:**
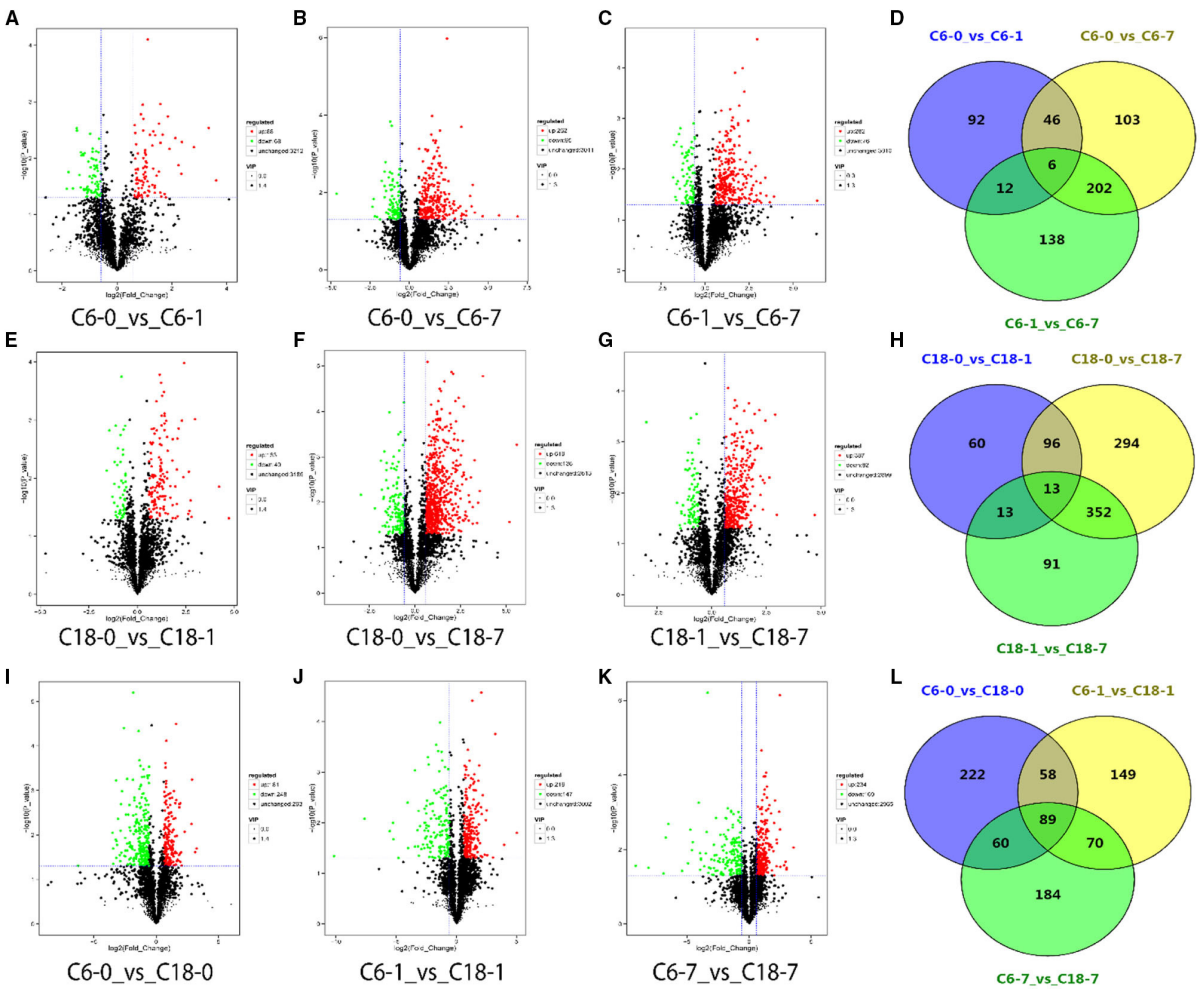
Volcano plots for differentially accumulated metabolites (DAMs) with the comparison of control with their time points. **(A–C)** DAMs from the cold-sensitive (C6) variety; **(E–G)** DAMs from the cold-tolerant (C18) variety; **(I–K)** DAMs between C6 and C18 at the same time points. Venn diagrams showing the shared and common DAMs for all time points **(D)** between the different time points in C6; **(H)** between the different time points in C18; **(L)** between the same time points in C6 and C18.

Differentially accumulated metabolites (DAMs) were screened with a screening standard of FC > 1.5, *P* < 0.05, and VIP > 1. To identify the DAMs of rapeseed under CS, we compared the metabolite numbers from the control condition to their stress level at each time point. Out of the total (3,368), we identified 156 DAMs from the comparison of C6-0 vs. C6-1, 357 DAMs from C6-0 vs. C6-7, 358 DAMs from C6-1 vs. C6-7, respectively (Figures 2A–C and Supplementary Table 4). For the CT-C18 variety, 182 DAMs from C18-0 vs. C18-1, 755 DAMs from C18-0 vs. C18-7, and 469 DAMs from C18-1 vs. C18-7 were detected, respectively (Figures 2E–G and Supplementary Table 4). Furthermore, we also analyze the DAMs by comparing both varieties at each time point. From these comparisons, we identified 429 DAMs from C6-0 vs. C18-0, 366 DAMs from C6-1 v C18-1, and 403 DAMs from C6-7 vs. C18-7 that were either upregulated or downregulated (Figures 2I–K and Supplementary Table 4).

Notably, we observed an increased number of DAMs over the stress time point in both varieties, which showed the active adaptation of the metabolites in response to CS (Supplementary Figure 2). Nevertheless, after 7 days of CS, more DAMs were detected in CT-C18 than CS-C6, which indicated the strong metabolic disturbance experienced by CT-C18. By analyzing the types of DAMs (either upregulated or downregulated) in both varieties at the different time points, it was observed that the CT-C18 variety responded rapidly to the stress based on the number of DAMs (Supplementary Figure 2A). However, when we compared the same time points of both varieties, after 1 day, the DAMs were notably higher at 7 days of stress (Supplementary Figure 2B). Furthermore, as the stress time increased, more DAMs were upregulated in CT-C18 than CS-C6, while a similar trend was observed for downregulated DAMs for both varieties (Supplementary Figure 2C). The comparison between CS-C6 and CT-C18 at the same time points indicated that, with prolonged treatment, an increasing number of upregulated DAMs were observed. Furthermore, a decreasing number of downregulated DAMs was detected after 1 day of treatment (Supplementary Figure 2D). In total, CS-C6 failed to accumulate the optimum number of DAMs, which may be related to CS sensitivity. On the contrary, CT-C18 performed better by accumulating a larger number and more upregulated DAMs throughout the stress conditions. This data also supports our finding that CS-C6 was sensitive to CS and CT-C18 showed significant tolerance to CS at metabolome levels.

### The Core Metabolites Responded to CS in Rapeseed Varieties

**Figure 3:**
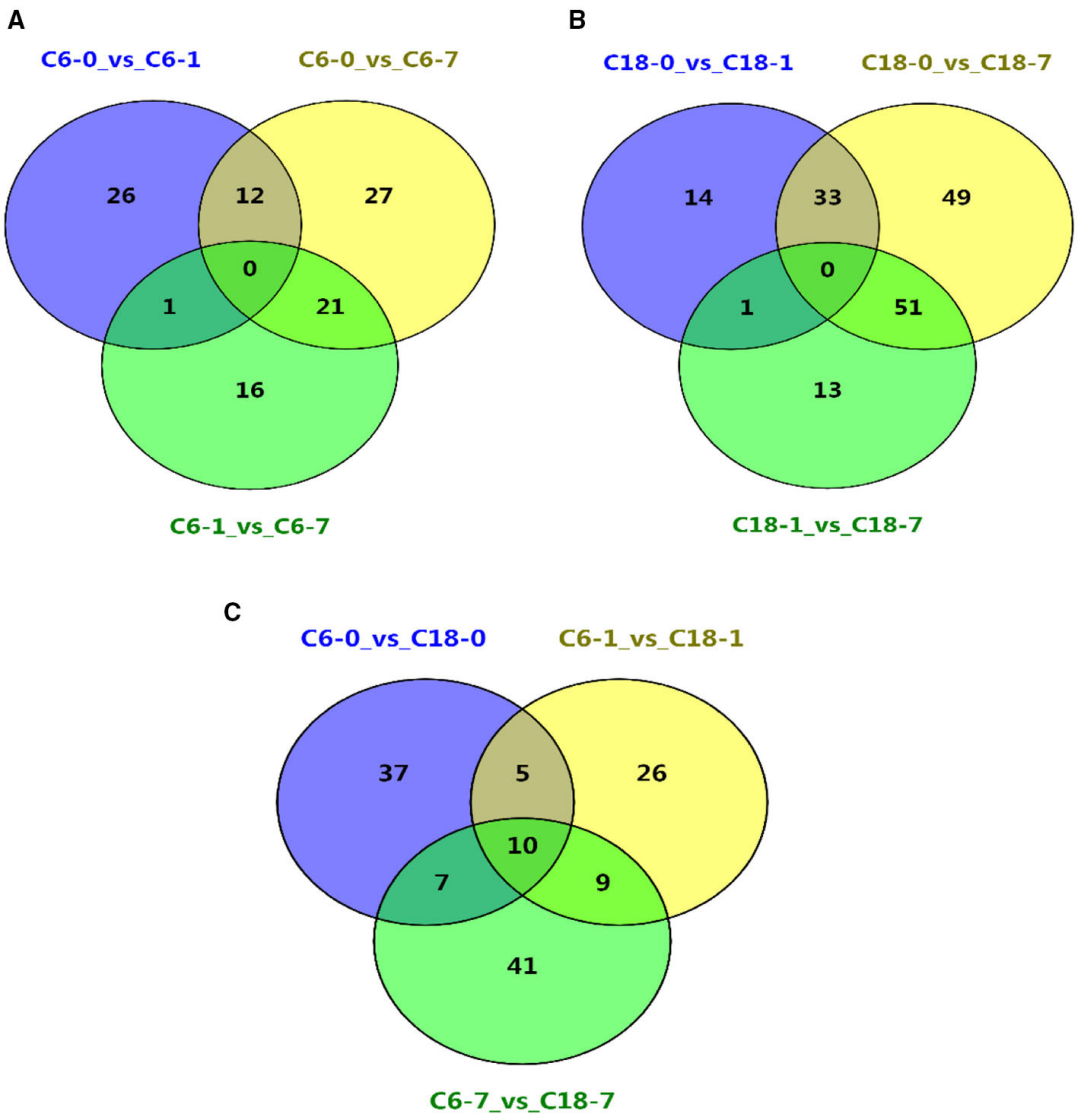
Venn diagrams showing the shared and commonly known DAMs for all time points **(A)** between the different time points in C6; **(B)** between the different time points in C18; **(C)** between the same time points in C6 and C18.

The common and specific DAMs were identified at different time points for both varieties under CS conditions (Figures 2D,H,L and Supplementary Table 5). However, some of these DAMs were known metabolites. Therefore, the named metabolites were compared using different comparisons. A considerable number of DAMs were commonly or specifically detected no matter if they were in comparison with one variety at a different time point (Figures 3A,B) or in comparison with both varieties at the same time point (Figure 3C and Supplementary Table 6). Interestingly, these results showed that CT-C18 accumulated a greater number of known and annotated DAMs compared to CS-C6 during the comparison of both varieties at the same time points (Figure 3C and Supplementary Table 6). The KEGG analysis of these DAMs disclosed that these metabolites mainly belonged to the following classes: amino acid metabolism, carbohydrate metabolism, biosynthesis of other secondary metabolites, membrane transport, lipid metabolism, metabolism of terpenoids and polyketides, metabolism of cofactors and vitamins, nucleotide metabolism, and energy metabolism (Supplementary Figures 3–5). Interestingly, there were no significantly enriched pathways in the comparisons of C6 1 vs. C6 7, C18 1 vs. C18 7, and C6 1 vs. C18 1 (Supplementary Figure 6).

Based on the above-discussed findings, the present study focused on the common DAMs (annotated) detected in comparing both varieties at the same time point (Figure 3C). The summary of these core DAMs is presented in Table 1. Interestingly, more upregulated metabolites were detected in the CT-C18 variety than CS-C6, indicating the positive role of such metabolites in CS tolerance and adaptation in rapeseed. Based on the KEGG annotation, 31 DAMs were involved in 19 pathways, including biosynthesis pathways, amino acid metabolism, carbohydrate metabolism, nucleotide metabolism, membrane transport, and energy metabolism (Supplementary Table 7).

**Table 1:** Information about important known DAMs with their compound ID, molecular formula, log_2_FC values, *p*-values, and type of regulations along with ko-ID identified by OPLS-DA model.

**S. no**.	**Metabolite ID**	**Metabolite name**	**Compound ID**	**Molecular formula**	**OPLS-DA model's VIP values**	**log_**2**_FC**	***P*** **-value**	**Regulated**	**ko-ID**
**(G1) 10 common known DAMs in “C6-0_vs_C18-0,” “C6-1_vs_C18-1,” and “C6-7_vs_C18-7”**									
1	Meta 665	3-Methylglutaric acid	C03761	C_6_H_10_O_5_	1.59/1.57/1.50	−2.58/−2.39/−2.88	0.0295/0.0309/0.0488	Down	N/A
2	Meta 1457	gamma-Tocotrienol	C14155	C_28_H_42_O_2_	1.39/1.49/1.47	0.84/1.10/1.11	0.0469/0.0119/0.0320	Up	ko00130
3	Meta 56	Imidazoleacetic acid	C02835	C_5_H_6_N_2_O_2_	1.55/1.53/1.60	−1.38/−1.47/−2.09	0.0207/0.0083/0.0106	Down	ko00340
4	Meta 1057	L-Gulonic gamma-lactone	C01040	C_6_H_10_O_6_	1.64/1.48/1.64	0.80/1.09/1.03	0.0016/0.0352/0.0018	Up	N/A
5	Meta 190	L-Kynurenine	C00328	C_10_H_12_N_2_O_3_	1.41/1.62/1.58	0.75/1.20/1.36	0.0382/0.0013/0.0034	Up	ko00380
6	Meta 1297	N,N'-Diacetylchitobiose	C01674	C_16_H_28_N_2_O_11_	1.67/1.63/1.61	−3.09/−2.32/−2.64	0.0043/0.0117/0.0141	Down	ko00520, ko02010
7	Meta 1529	Oxethazaine	C12552	C_28_H_41_N_3_O_3_	1.67/1.56/1.52	−1.88/−1.78/−1.94	0.0005/0.0199/0.0322	Down	N/A
8	Meta 958	Phenyllactic acid	C01207	C_9_H_10_O_5_	1.54/1.52/1.64	1.03/1.46/1.35	0.0328/0.0203/8.73E-05	Up	N/A
9	Meta 1678	Squalene	C00751	C_30_H_50_	1.65/1.61/1.61	−2.89/−3.24/−2.79	0.0080/0.0142/0.0145	Down	ko00100, ko00909
10	Meta 908	Trehalose	C01083	C_12_H_22_O_11_	1.56/1.56/1.65	1.20/1.26/1.27	0.0268/0.0121/0.0001	Up	ko00500, ko02010
**(G2) 7 common known DAMs in “C6-0_vs_C18-0” and “C6-7_vs_C18-7”**									
11	Meta 217	4-Hydroxyphenylpyruvate	C01179	C_9_H_8_O_4_	1.38/1.37	−0.66/−1.01	0.0423/0.0452	Down	ko00400, ko01210, ko00261, ko00130, ko00950, ko01230, ko00350
12	Meta 1149	4-Methylumbelliferyl beta-D-glucuronide	91553^a^	C_16_H_16_O_9_	1.66/1.54	−1.45/−1.01	0.0053/0.0270	Down	N/A
13	Meta 1043	Adenosine 3′,5′-cyclic phosphate (cAMP)	6076^a^	C_10_H_12_N_5_O_6_P	1.54/1.64	1.92/1.76	0.0402/0.0010	Up	N/A
14	Meta 1197	Aminopterin	169371^a^	C_19_H_20_N_8_O_5_	1.45/1.39	−0.59/0.66	0.0336/0.0368	Down	N/A
15	Meta 1762	Doxorubicin	C01661	C_27_H_29_NO_11_	1.60/1.56	−1.19/−1.01	0.0066/0.0049	Down	N/A
16	Meta 338	N-.alpha.-Acetyl-L-arginine	67427^a^	C_8_H_16_N_4_O_3_	1.59/1.46	−0.79/−0.64	0.019302849/ 0.026369916	Down	N/A
17	Meta 800	N-Acetylaspartylglutamate (NAAG)	188803^a^	C_11_H_16_N_2_O_8_	1.64/1.55	0.66/0.79	0.0013/0.0229	Up	N/A
**(G3) 5 common known DAMs in “C6-0_vs_C18-0” and “C6-1_vs_C18-1”**									
18	Meta 1215	Acetylcarnitine	7045767^a^	C_9_H_17_NO_4_	1.56/1.48	0.59/1.50	0.0241/0.0127	Up	N/A
19	Meta 291	Alpha-D-Glucose	C00267	C_6_H_12_O_6_	1.62/1.49	1.82/0.76	0.0024/0.0176	Up	ko00520, ko00052, ko00051, ko01200, ko00010
20	meta 319	Altretamine	D02841	C_9_H_18_N_6_	1.68/1.60	−3.24/−2.36	0.0005/0.0145	Down	N/A
21	Meta 481	Deoxyadenosine	C00559	C_10_H_13_N_5_O_3_	1.44/1.52	1.14/1.34	0.0372/0.0099	Up	ko00230
22	Meta 292	Leu-Ala	6992295^a^	C_9_H_18_N_2_O_3_	1.63/1.62	−1.39/−0.88	0.0139/0.0019	Down	N/A
**(G4) 9 common known DAMs in “C6-1_vs_C18-1” and “C6-7_vs_C18-7”**									
23	Meta 959	Cyclopentolate	C06932	C_17_H_25_NO_3_	1.62/1.54	−3.17/−3.95	0.0059/0.0352	Down	N/A
24	Meta 1522	Cytidine 5'-diphosphocholine (CDP-choline)	25202509^a^	C_14_H_25_N_4_O_11_${\text{P}}_{2}^{-}$	1.52/1.56	0.80/0.64	0.0133/0.0051	Up	N/A
25	Meta 722	Dimethyl 4,4-o-Phenylene-Bis (3-Thiophanate)	3032791^a^	C_12_H_14_N_4_O_4_S_2_	1.60/1.54	0.87/0.75	0.0032/0.0074	Up	N/A
26	Meta 216	DL-Vanillylmandelic acid	C05584	C_9_H_10_O_5_	1.56/1.55	0.95/1.22	0.0107/0.0092	Up	ko00350
27	Meta 26	Larixinic Acid	8369^a^	C_6_H_6_O_3_	1.59/1.49	0.96/0.89	0.0229/0.0158	Up	N/A
28	Meta 1611	Malvidin 3-O-glucoside cation	C12140	C_23_H_25_O_12_	1.60/1.47	1.68/1.39	0.0173/0.0207	Up	N/A
29	Meta 390	N-Acetyl-L-phenylalanine	C03519	C_11_H_13_NO_3_	1.57/1.55	0.86/1.24	0.0108/0.0154	Up	ko00360
30	Meta 1900	Pristimerin	C08633	C_30_H_40_O_4_	1.52/1.53	−1.34/−1.19	0.0390/0.0189	Down	N/A
31	Meta 983	Tyr-Asp	CHEBI:141455^b^	C_13_H_16_N_2_O_6_	1.54/1.57	1.04/1.32	0.0351/0.0067	Up	N/A

*OPLS-DA, orthogonal projections to latent structures-discriminant analysis; VIP, variable importance in projection; log_2_FC, log_2_ of fold change values; P-value, p-value of the student's t-test*.

a *PubChem CID*;

b *ChEBI ID*.

### Analysis Based on Transcriptome Data of C6 and C18 Under CS Conditions

**Figure 4:**
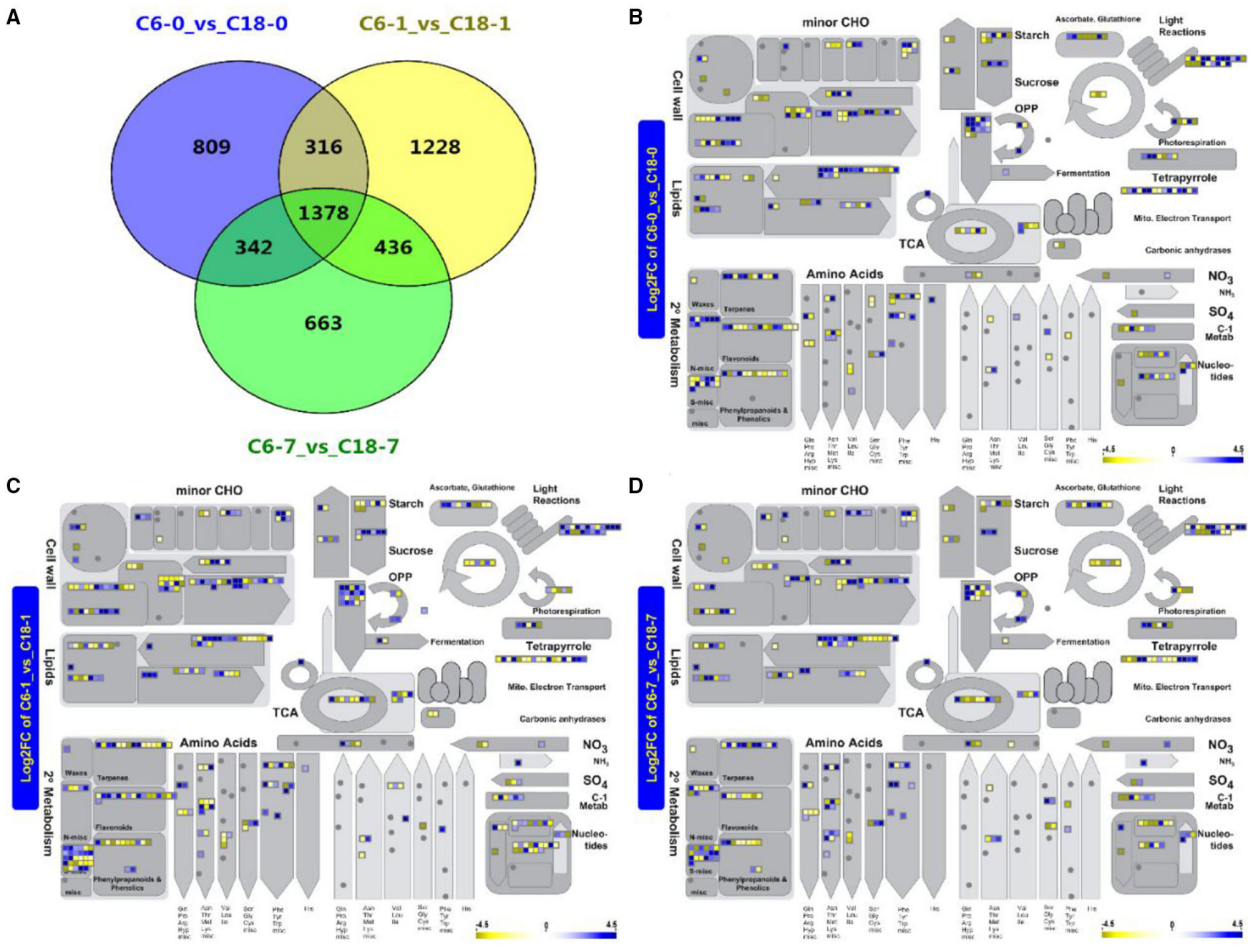
**(A)** Venn diagram showing the shared and common differentially expressed genes (DEGs) for the same time points between C6 and C18 varieties. **(B–D)** Metabolism overview of all DEGs at 0 (CK), 1, and 7 days of stress between both cultivars revealed by the MapMan pathway annotator, respectively. **(B)** A total of 2,725 of the 2,845 data points were mapped (some of the data points may be mapped multiple times to different bins), and 363 genes are visible in data points; **(C)** A total of 3,297 of the 3,358 data points were mapped (some of the data points may be mapped multiple times to different bins) and 449 genes are visible in data points; **(D)** A total of 2,713 of the 2,829 data points were mapped (some of the data points may be mapped multiple times to different bins) and 360 genes are visible in data points. Boxes in a blue or yellow color mean significantly up- and downregulated genes, respectively. CHO, carbohydrate; OPP, oxidative pentose phosphate; TCA, tricarboxylic acid.

To dissect how the two rapeseed varieties differed in their response to CS at the transcript level, the global transcriptome profiles of CT-C18 and CS-C6 were constructed using a deep RNA-seq strategy (BioProject ID. PRJNA596550). Samples were obtained after 0, 1, and 7 days of exposure to CS. The same was done with the samples for the metabolome analysis. The standards of log_2_FC > 4, adjusted *p* < 0.001, and FDR <0.001 were used to recognize the DEGs in response to CS. From the transcriptome analysis, we detected 2,845 DEGs from the comparison of C6-0 vs. C18-0, 3,358 DEG from C6-1 vs. C18-1, and 2,819 DEG from C6-7 vs. C18-7 (Figure 4A and Supplementary Table 8). To predict the functional insights of all DEGs, the MapMan pathway annotator was used for the metabolism overview of the DEGs. It was found that rapeseed responded to CS depending on the genetic background (Figures 4B–D), which is supported by previous reports (Lei et al., 2018; Zhang C. et al., 2019; Wrucke et al., 2020). A similar expression trend for up- and downregulated DEGs was mainly detected in the secondary metabolism, amino acids, lipids, cell wall, minor CHO, and light reaction pathways during the three comparisons. More genes were enriched in the secondary metabolism, amino acids, lipids, and cell wall pathways (Figures 4B–D). Interestingly, most of the pathways were found to be enriched with upregulated genes at 1 and 7 days of stress (Figures 4C,D). Mainly, secondary metabolism, amino acids, lipids, and cell wall pathways exhibited a greater number of DEGs. These outcomes indicate that these DEGs and associated metabolic pathways could help rapeseed adapt to CS conditions. The results here showed that the step-wise overview of metabolism pathways in rapeseed gave a clear presentation of the specific pathways in rapeseed that functioned at different time points upon CS treatment (Figure 4).

### Network Analysis of DAMs and DEGs in Response to CS

To understand the regulatory networks of DAMs between the CS-C6 and CT-C18 varieties, we carried out a correlation test between the core DAMs and DEGs in the three groups (C6-0 vs. C18-0, C6-1 vs. C18-1, and C6-7 vs. C18-7) (Table 1). These DEGs were subjected to PCC analysis, and screening criteria were PCC > 0.8. Interaction networks were organized between the DAMs that were annotated by the KEGG database. Therefore, to get insights into the type and strength of correlation among DAMs and DEGs, an interaction network was performed among four different groups at different time points in both varieties (see Table 1 for groups setting; G1-G4).

**Figure 5:**
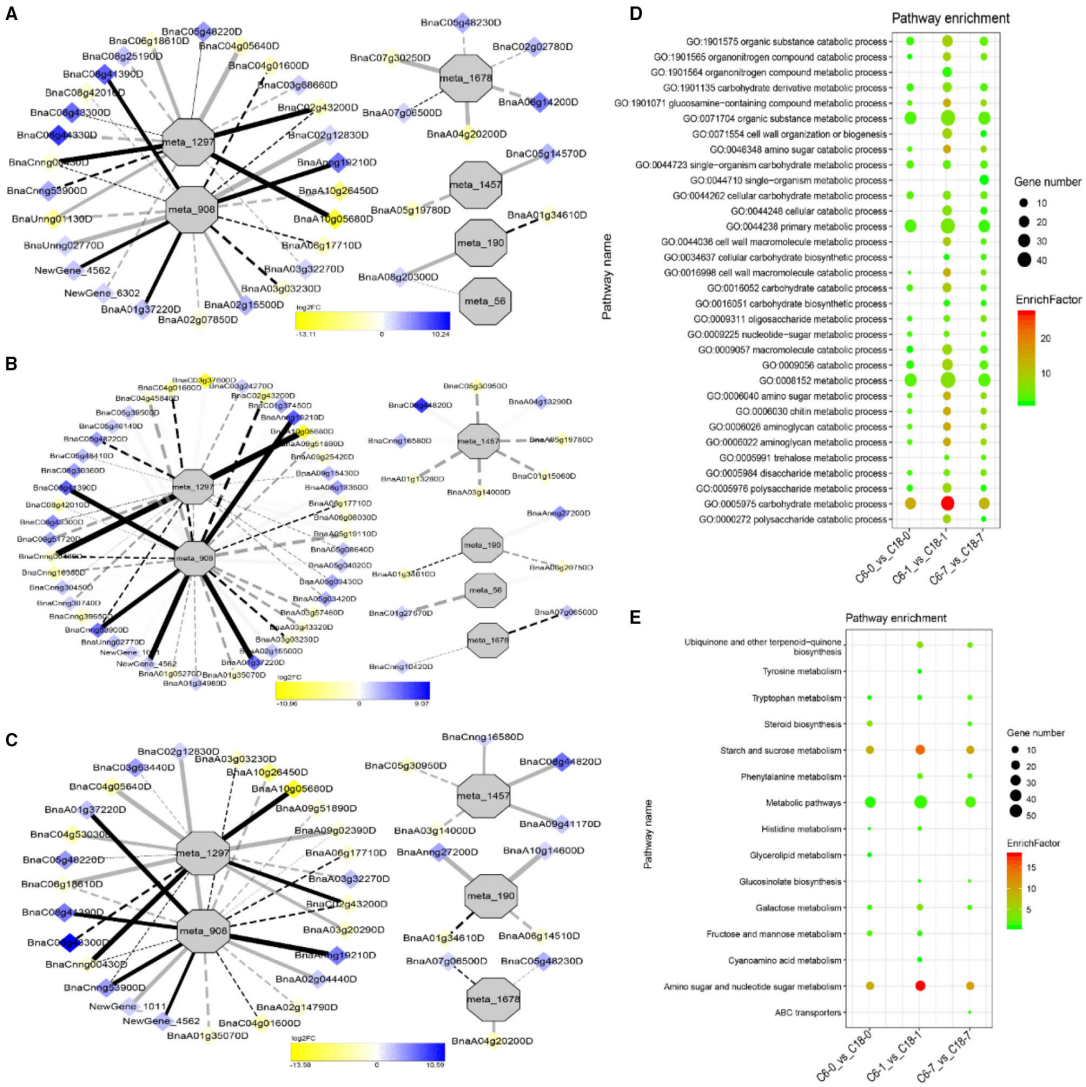
Correlation network analysis between DAMs and regulatory genes related to cold stress (CS). **(A)** Analysis of C6-0 vs. C18-0, **(B)** analysis of C6-1 vs. C18-1, and **(C)** analysis of C6-7 vs. C18-7. Log_2_FC of genes, in response to CS, are indicated in heatmap colors ranging from yellow (downregulated) to blue (upregulated); log_2_FC values are indicated in the legend; the width of the connecting lines indicating the strength of the correlation; solid lines indicating the positive correlation and dashed lines indicating the negative correlation; black lines indicating the common genes and gray lines indicating the specific genes accumulated at that time point. **(D,E)** Scatter plots for the gene ontology-biological process (GO-BP) enrichment analysis and the Kyoto Encyclopedia of Genes and Genomes (KEGG) pathway enrichment analysis of DEGs from G1, respectively. Only the highly enriched and most relevant terms and pathways are shown in the plots (*Q* < 0.05.) –log^10^ (*Q*) for enrichment factor.

**Figure 6:**
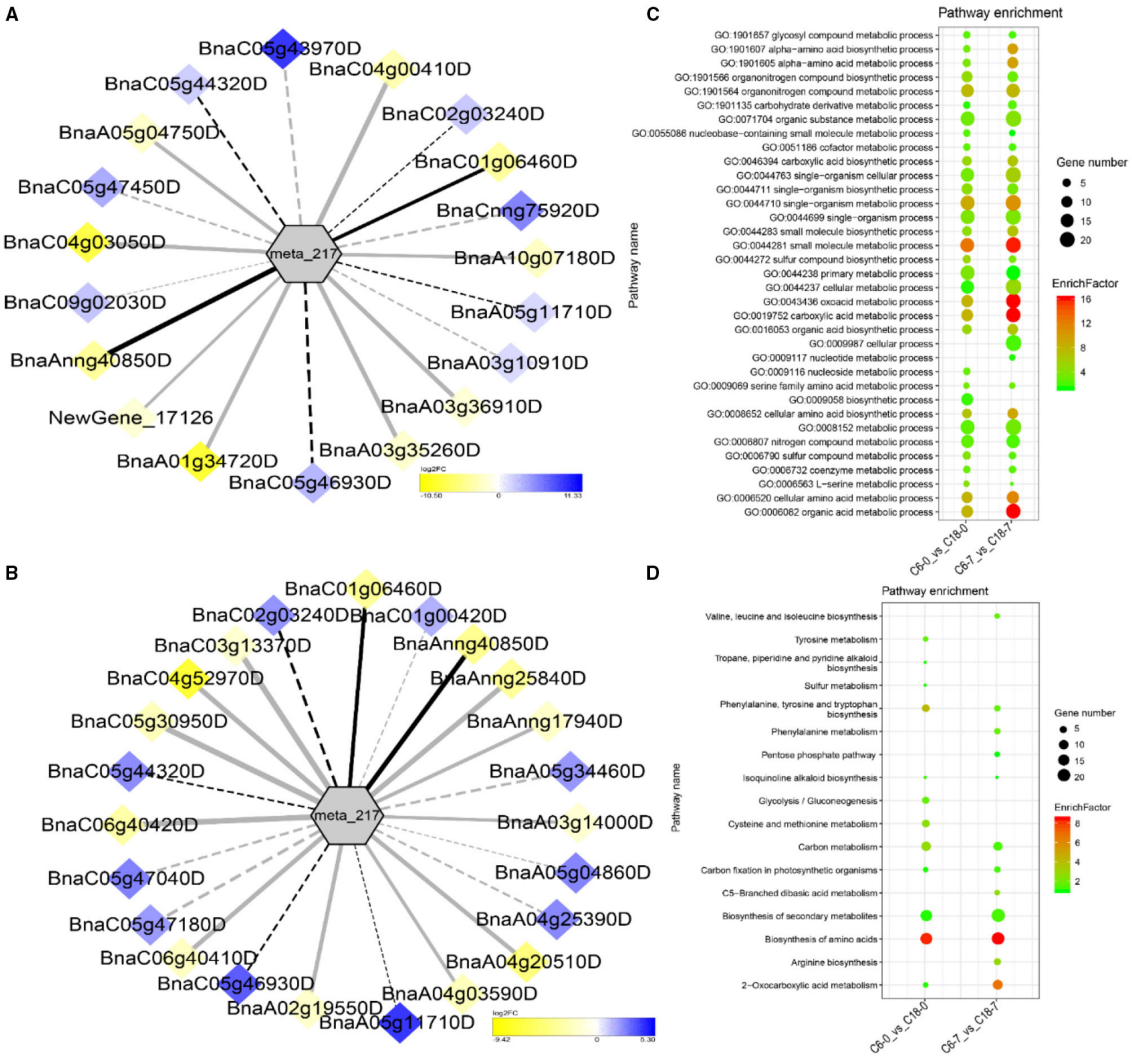
Correlation network analysis between DAMs and regulatory genes related to CS. **(A)** Analysis of C6-0 vs. C18-0 and **(B)** analysis of C6-7 vs. C18-7. Log_2_FC of genes, in response to CS, are indicated in heatmap colors ranging from yellow (downregulated) to blue (u-regulated); log_2_FC values are indicated in the legend; the width of the connecting lines indicating the strength of the correlation; solid lines indicating the positive correlation and dashed lines indicating the negative correlation; black lines indicating the common genes and gray lines indicating the specific genes accumulated at that time point. **(C,D)** Scatter plots for the GO-BP enrichment analysis and KEGG pathway enrichment analysis of DEGs from G2, respectively. Only the highly enriched and most relevant terms and pathways are shown in the plots (*Q* < 0.05.) –log^10^ (*Q*) for enrichment factor.

**Figure 7:**
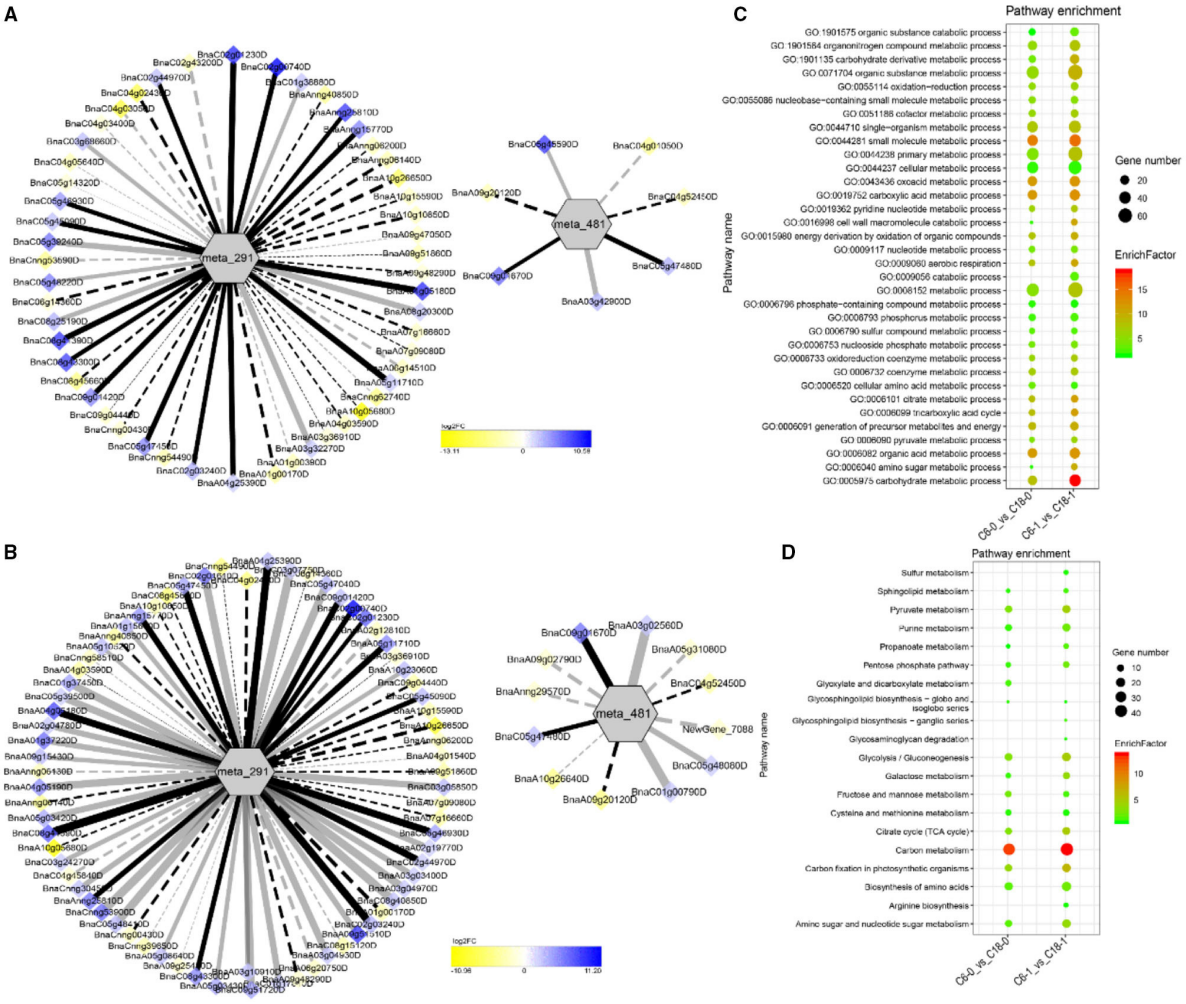
Correlation network analysis between DAMs and regulatory genes related to CS. **(A)** Analysis of C6-0 vs. C18-0 and **(B)** analysis of C6-1 vs. C18-1. Log_2_FC of genes, in response to CS, are indicated in heatmap colors ranging from yellow (downregulated) to blue (upregulated); log_2_FC values are indicated in the legend; the width of the connecting lines indicating the strength of the correlation; solid lines indicating the positive correlation and dashed lines indicating the negative correlation; black lines indicating the common genes and gray lines indicating the specific genes accumulated at that time point. **(C,D)** Scatter plots for the GO-BP enrichment analysis and KEGG pathway enrichment analysis of DEGs from G3, respectively. Only the highly enriched and most relevant terms and pathways are shown in the plots (*Q* < 0.05.) –log^10^ (*Q*) for enrichment factor.

**Figure 8:**
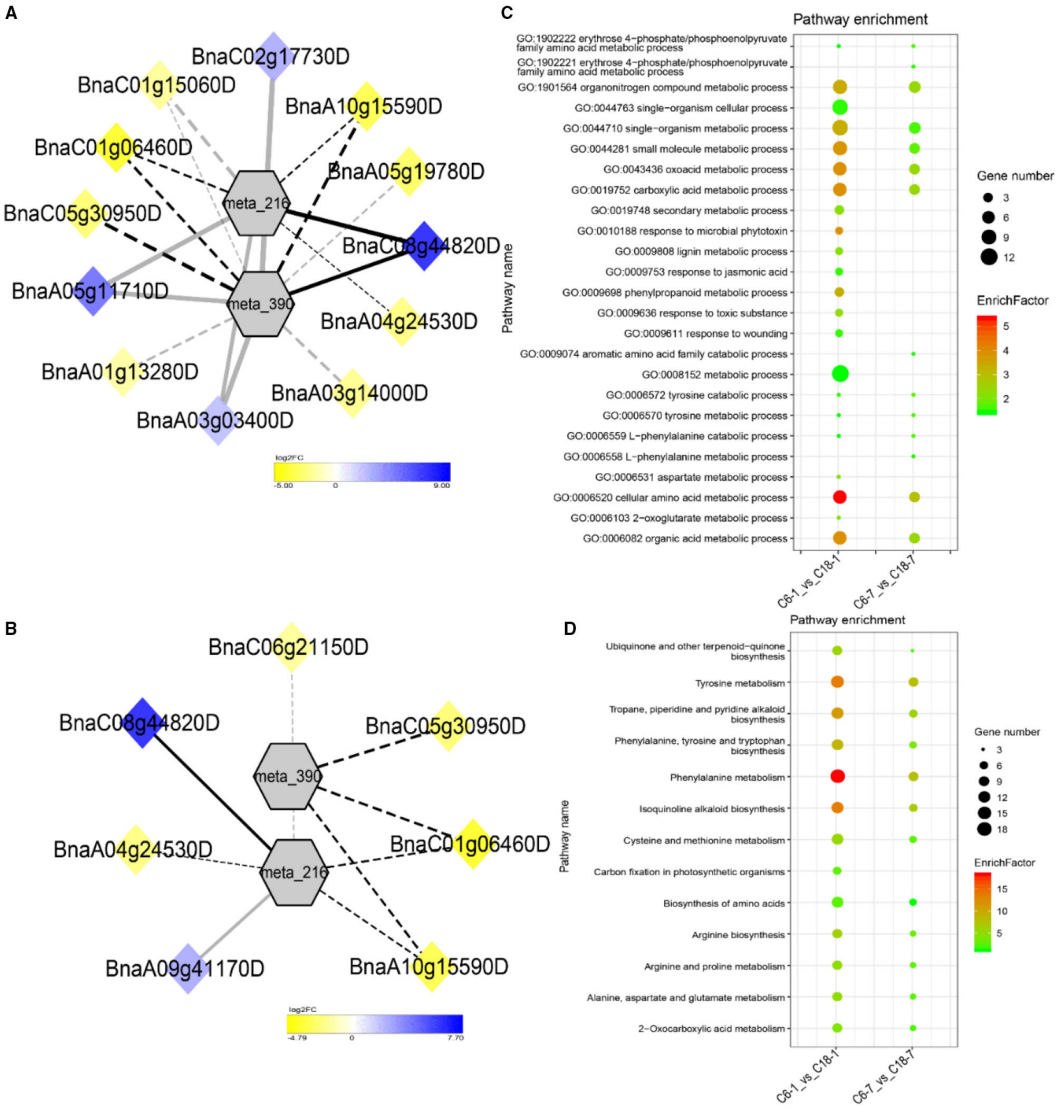
Correlation network analysis between DAMs and regulatory genes related to CS. **(A)** Analysis of C6-1 vs. C18-1 and **(B)** analysis of C6-7 vs. C18-7. Log_2_FC of genes, in response to CS, are indicated in heatmap colors ranging from yellow (downregulated) to blue (upregulated); log_2_FC values are indicated in the legend; the width of the connecting lines indicating the strength of the correlation; solid lines indicating the positive correlation and dashed lines indicating the negative correlation; black lines indicating the common genes and gray lines indicating the specific genes accumulated at that time point. **(C,D)** Scatter plots for the GO-BP enrichment analysis and KEGG pathway enrichment analysis of DEGs from G4, respectively. Only the highly enriched and most relevant terms and pathways are shown in the plots (*Q* < 0.05.) –log^10^ (*Q*) for enrichment factor.

**Figure 9:**
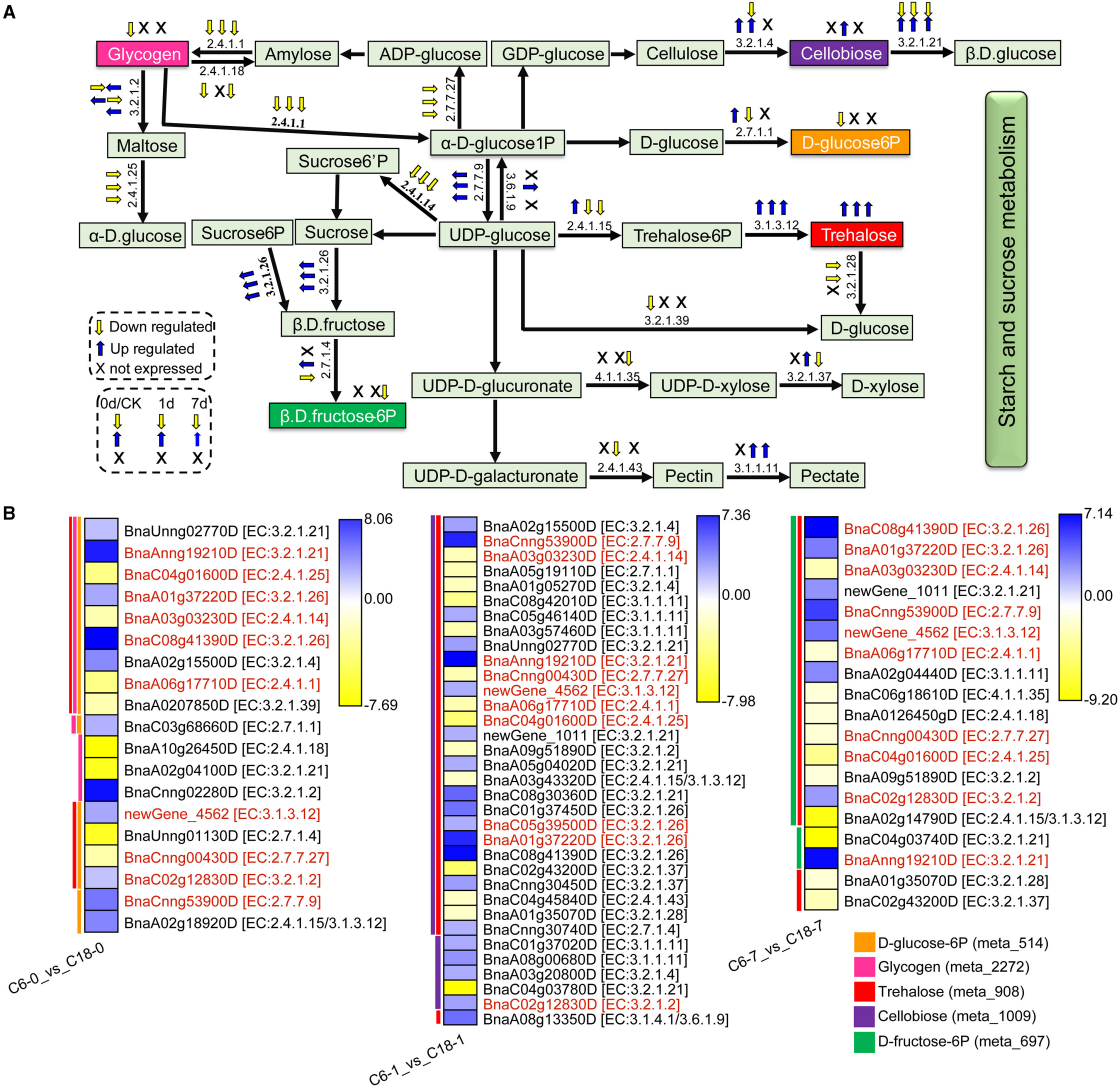
The proposed layout of the starch and sucrose metabolism pathway in response to CS in C6 and C18 cultivars. **(A)** Layout showing the DEGs involved in starch and sucrose metabolism. Colored boxes indicate the metabolites observed in this pathway. This pathway shows only the DEGs that have been correlated with the metabolites from the combined analysis of transcriptomes and metabolomes. The yellow and blue arrows indicate the expression pattern (up-/downregulated) of DEGs based on the three comparisons, i.e., C6-0 vs. C18-0, C6-1 vs. C18-1, and C6-7 vs. C18-7, whereas “X” indicates not expressed at specific time points. **(B)** Heatmap showing the expression of DEGs corresponding to “A.” Red text color for gene names indicating the shared/common DEGs. Colored bars on the left side of each heatmap showing the metabolites correlated with DEGs based on Pearson correlation coefficient (PCC) analysis. The enzyme commission (EC) numbers are as follows: 2.4.1.1, glycogen phosphorylase; 2.4.1.14, sucrose-phosphate synthase; 2.4.1.15/3.1.3.12, trehalose 6-phosphate synthase/phosphatase; 2.4.1.18, 1,4-alpha-glucan branching enzyme 2-2; 2.4.1.25, 4-alpha-glucanotransferase; 2.4.1.43, alpha-1,4-galacturonosyltransferase; 2.7.1.1, hexokinase; 2.7.1.4, fructokinase; 2.7.7.9, UTP–glucose-1-phosphate uridylyltransferase; 2.7.7.27, glucose-1-phosphate adenylyltransferase; 3.1.1.11, pectinesterase; 3.1.3.12, trehalose 6-phosphate phosphatase; 3.2.1.2, beta-amylase; 3.2.1.4, endoglucanase; 3.2.1.21, beta-glucosidase; 3.2.1.26, beta-fructofuranosidase; 3.2.1.28, alpha, alpha-trehalase; 3.2.1.37, beta-D-xylosidase 4; 3.2.1.39, glucan endo-1,3-beta-glucosidase 5/6; 3.1.4.1/3.6.1.9, ectonucleotide pyrophosphatase/ phosphodiesterase family member 1/3; 4.1.1.35, UDP-glucuronate decarboxylase.

The interaction network for G1 was performed between 6 DAMs and 41 DEGs from C6-0 vs. C18-0 (Figure 5A), 6 DAMs and 67 DEGs from C6-1 vs. C18-1 (Figure 5B), and 5 DAMs and 44 DEGs from C6-7 vs. C18-7 (Figure 5C, and Supplementary Table 9). The network showed that meta_908 (Trehalose) and meta_1297 (N,N'-Diacetylchitobiose) were clustered together and shared 19 common DEGs. However, these common DEGs showed diverse expression patterns in the three comparisons. Meanwhile, meta_56 (Imidazoleacetic acid), meta_190 (L-Kynurenine), meta_1457 (gamma-Tocotrienol), and meta_1678 (Squalene) were clustered separately (Figure 5A). The interaction network for G2 was carried out between 1 DAM and 19 DEGs from C6-0 vs. C18-0 (Figure 6A) and 23 DEGs from C6-7 vs. C18-7 (Figure 6B). There were 5 common DEGs (Supplementary Table 10). The results showed that meta_217 (4-Hydroxypheny lpyruvate) was correlated with DEGs at 0 and 7 days of CS. The interaction network for G3 was visualized between 2 DAMs and 59 DEGs for C6-0 vs. C18-0 (Figure 7A) and 85 DEGs for C6-1 vs. C18-1 (Figure 7B). There were 39 DEGs shared between these two networks. Meta_291 (Alpha-D-Glucose) and meta_481 (Deoxyadenosin) were correlated with more DEGs at 1 day of stress than that of 0 days (Supplementary Table 11). Interaction network for G4 was carried out among 2 DAMs and 18 DEGs for C6-1 vs. C18-1 (Figure 8A) and 9 DEGs for C6-7 vs. C18-7 (Figure 8B); among which, 5 DEGs were common between these two groups. The network showed that meta_216 (DLVanillylmandelic acid) and meta_390 (N-Acetyl-L-phenylalanine) were clustered and associated with common and specific DEGs (Supplementary Table 11). Overall, our results showed strong and positive correlations between different DAMs and DEGs (involved in the same metabolic pathways), thus, contributing to CS tolerance and adaption in rapeseed.

### GO and KEGG Analysis of DEGs Detected by Correlation Analysis

Differentially expressed genes correlated with G1-G4 (see Table 1 for group settings) were further subjected to GO-biological process (BP) category analysis to determine the specific GO-BP terms related to DAMs under CS conditions. As shown in Figure 5D, the highly enriched DEGs are associated with the organic substance metabolic process (GO:0071704), primary metabolic process (GO:0044238), and metabolic process (GO:0008152). Most of the terms existed throughout the stress and only a few terms were specific to one time point. For instance, the carbohydrate metabolic process (GO:0005975) was highly significant and enriched at 1 day as compared to 0 and 7 days of CS. In comparison, some terms were explicitly enriched at 1 and 7 days, like the organonitrogen compound metabolic process (GO:1901564) and the single-organism metabolic process (GO:0044710). Similarly, DEGs correlated with meta_217 (Table 1, G2) were highly enriched in the same terms at 0 or 7 days of CS. Among these, the oxoacid metabolic process (GO:0043436), carboxylic acid metabolic process (GO:0019752), and organic acid metabolic process (GO:0006082) were more highly enriched at 7 days than that of 1 day (Figure 6C). The findings of G3 indicated that the carbohydrate metabolic process (GO:0005975) was significantly enriched at 1 day of CS treatment (Figure 7C). However, the enriched terms of DEGs from the two comparisons (C6-1 vs. C18-1 and C6-7 vs. C18-7) were totally different. Only the cellular amino acid metabolic process (GO:0006520) was highly enriched at 1 day (Figure 8C). Given the above observation, it was discovered that the enriched GO-BP terms were mainly the carbohydrate metabolic process, cellular amino acid metabolic process, organic acid metabolic process, carboxylic acid metabolic process, and oxoacid metabolic process. Taken together, the GO enrichment analysis in this study provided important guidance to identify DEGs that functioned in key pathways that may directly or indirectly involve in the CS response of rapeseed.

To further exploit the enriched metabolic pathways related to CS tolerance and adaptation in rapeseed, DEGs from G1 to G4 (Table 1) were subjected to KEGG enrichment analysis. The results of G1 showed that amino sugar and nucleotide sugar metabolism was mostly enriched throughout the CS at 0, 1, and 7 days. A similar trend was observed for starch and sucrose metabolism (Figure 5E). The results of G2 revealed that the biosynthesis of amino acids was the most significant and enriched pathway existing in two time points, i.e., 1 and 7 days of CS. Notably, 2–Oxocarboxylic acid metabolism was specifically enriched at 7 days of CS (Figure 6D). The results of G3 indicated that carbon metabolism was enriched at 0 or 1 day of CS, which was consistent with the corresponding GO-BP analysis (Figure 7D). Similarly, phenylalanine metabolism, tyrosine metabolism, isoquinoline alkaloid biosynthesis, tropane, piperidine, and pyridine alkaloid biosynthesis were enriched terms in G4 at 1 day of CS (Figure 8D). As a result, we discovered that the KEGG and GO-BP analyses were mainly matched with each other, generating two terms (amino acid metabolisms and carbohydrate metabolisms) associated with CS responses in both rapeseed varieties. These comparable findings of GO-BP and KEGG enrichment analyses showed that mainly amino acid and carbohydrate metabolisms could play a crucial role in the CS tolerance and adaptation of rapeseed.

### Starch and Sucrose Metabolism in Response to CS

Based on the GO and KEGG enrichment analyses, many DAMs and DEGs of rapeseed were correlated with starch and sucrose metabolism under CS conditions. In total, 5 DAMs were correlated with 19, 34, and 19 DEGs from the comparisons of C6-0 vs. C18-0, C6-1 vs. C18-1, and C6-7 vs. C18-7, respectively. Figure 9A shows the proposed general layout of starch and sucrose metabolism and the relative changes of 5 DAMs during CS treatment based on the three comparisons. Trehalose (meta_908) was the only metabolite that accumulated throughout the stress, followed by cellobiose (meta_1009), which accumulated only at 1 day of treatment, suggesting that CS-C6 should keep a higher level of trehalose and cellobiose than that of CT-C18. Meanwhile, glycogen (meta_2272), D-glucose-6P (meta_514), and D-fructose-6P (meta_697) were downregulated at a different time point of the treatment, indicating that CT-C18 should keep a higher level of glycogen, D-glucose-6P, and D-fructose-6P than that of C6. These outcomes indicate that the higher contents of different sugar-related compounds could significantly contribute to CS tolerance in rapeseed.

The expression patterns of the DEGs involved in the starch and sucrose metabolism pathway were summarized in the heatmap (Figure 9B). These DEGs were correlated with one to three of the 5 mentioned DAMs and revealed diverse expression patterns in both varieties. For instance, D-glucose-6P was one of the major products of starch and sucrose metabolism that were correlated with 16 DEGs. The three DEGs directly related to the glucose branch showed lower expression in C6, subsequently leading to a lower accumulation of D-glucose-6P in CS-C6. However, the DEGs that functioned in other branches were up- or downregulated in the comparisons. Besides D-glucose-6P, no significant difference was observed for the other final product contents of the starch and sucrose metabolism pathway within the two rapeseed varieties. These results suggested that the glucose branch was strongly improved in CT-C18 and may contribute to the CS tolerance of rapeseed.

Notably, 10 DEGs (common/shared) were detected by three comparisons (Figure 9B). Among them, six DEGs exhibited higher expression level in CS-C6, encoding beta-glucosidase (BnaAnng19210D), beta-fructofuranosidase (BnaA01g37220D or BnaC08g41390D), trehalose-6-phosphate phosphatase (newGene_4562), beta-amylase (BnaC02g12830D), and UTP–glucose-1-phosphate uridylyltransferase (BnaCnng53900D) enzymes. Furthermore, 4 DEGs exhibited higher expression level in C18, which encoded 4-alpha-glucanotransferase (BnaC04g01600D), sucrose-phosphate synthase (BnaA03g03230D), glycogen phosphorylase (BnaA06g17710D), and glucose-1-phosphate adenylyltransferase (BnaCnng00430D) enzymes. These enzymes led to the strong CS-induced accumulation of different forms of glucose, fructose, trehalose, and maltose. Most of the genes responsible for the biosynthesis of these carbohydrates were upregulated in both varieties under CS and showed greater changes in CT-C18 than CS-C6 at 1 or 7 days after exposure to CS (Figure 9B). This suggests that CT-C18 had performed better in accumulating some carbohydrates and, thus, showed an improved CS tolerance level compared to the CS-C6 variety. On the contrary, the lower level of these molecules might lead to sensitivity under CS conditions.

### Phenylalanine Metabolism in Response to CS

**Figure 10:**
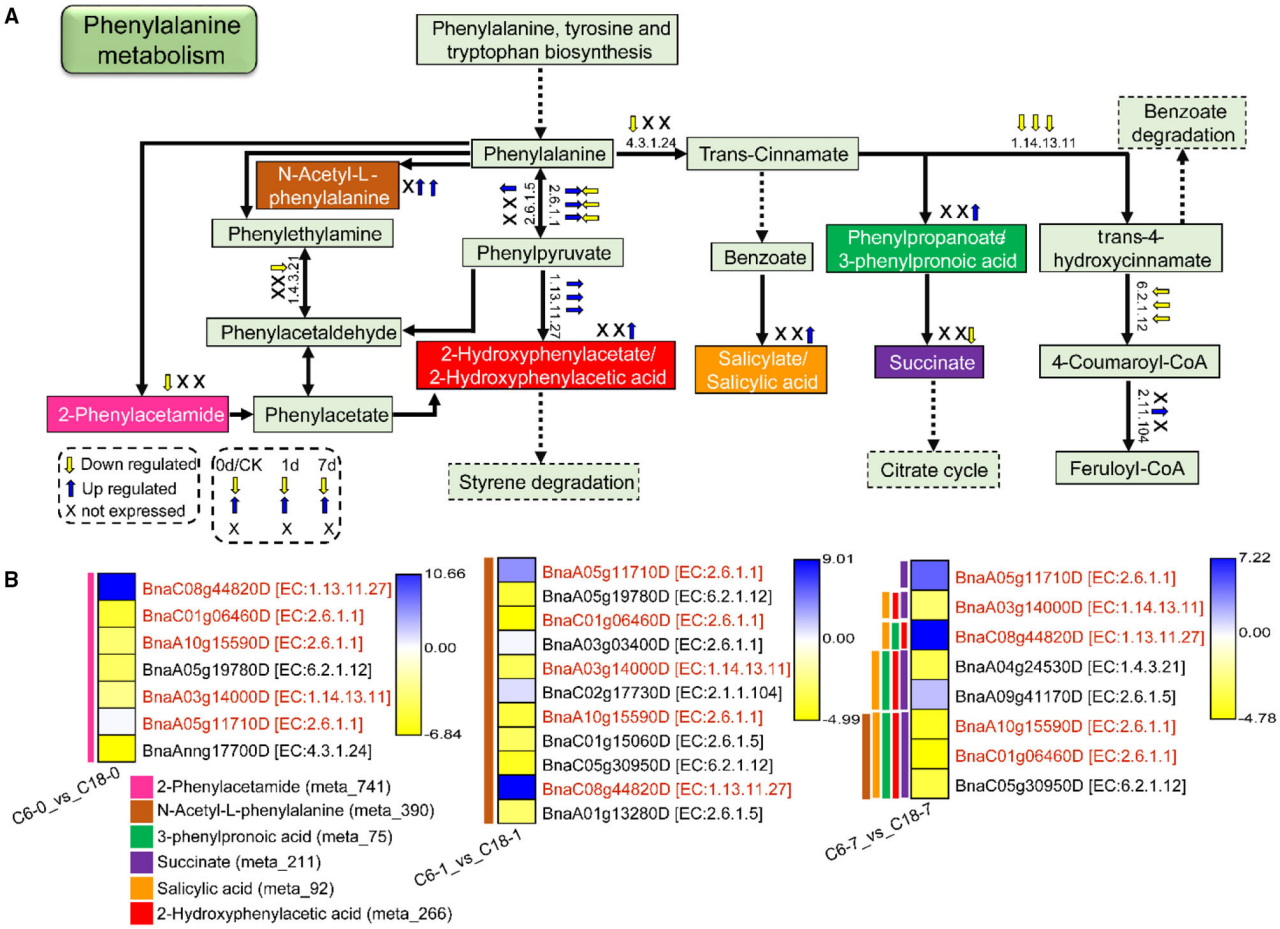
The proposed layout of the phenylalanine metabolism pathway in response to CS in C6 and C18 cultivars. **(A)** Layout showing the DEGs involved in the phenylalanine metabolism. Colored boxes are indicating the metabolites observed in this pathway. This pathway shows only the DEGs that have been correlated with the metabolites from the combined analysis of transcriptomes and metabolomes. Dotted lines show the omission of some reactions. The yellow and blue arrows indicate the expression pattern (up-/downregulated) of DEGs based on the three comparisons, i.e., C6-0 vs. C18-0, C6-1 vs. C18-1, and C6-7 vs. C18-7, whereas “X” indicates not expressed at specific time points. **(B)** Heatmap showing the expression of DEGs corresponding to “A.” Red text color for gene names indicating the shared/common DEGs. Colored bars on the left side of each heatmap showing the metabolites correlated with DEGs based on PCC analysis. The EC numbers are as follows: 1.4.3.21, primary-amine oxidase; 1.13.11.27, 4-hydroxyphenylpyruvate dioxygenase; 1.14.13.11, trans-cinnamate 4-monooxygenase; 2.6.1.1, aspartate aminotransferase, chloroplastic/cytoplasmic; 2.6.1.5, tyrosine aminotransferase; 2.1.1.104, caffeoyl-CoA O-methyltransferase; 4.3.1.24, phenylalanine ammonia-lyase; 6.2.1.12, coumarate–CoA ligase.

It was found that phenylalanine metabolism was significantly enriched in both metabolome and transcriptome data. In total, 6 DAMs were correlated with 7, 11, and 8 DEGs detected from C6-0 vs. C18-0, C6-1 vs. C18-1, and C6-7 vs. C18-7, respectively. Figure 10A shows the schematic layout of phenylalanine metabolism and the relative changes of the six DAMs in both varieties under CS conditions. Notably, only two DAMs, i.e., 2-Phenylacetamide and N-Acetyl-L-phenylalanine, were accumulated at 0 and 1 day while 2-Hydroxyphenylacetic acid, N-Acetyl-L-phenylalanine, 3-Phenylpropanoic acid, and salicylic acid were upregulated, and succinate was downregulated at 7 days (Figure 10).

The expression patterns of DEGs involved in the phenylalanine metabolism pathway were presented in the heatmap (Figure 10B). Particularly, 5 DEGs (common) were detected by three comparisons (Figure 8B). Among these, two genes were upregulated and encoding 4-hydroxyphenylpyruvate dioxygenase (BnaC08g44820D) and aspartate aminotransferase (BnaA05g11710D)-mitochondrial chloroplastic/cytoplasmic enzymes, whereas three genes were downregulated, among which two genes were encoding aspartate aminotransferase (BnaC01g06460D and BnaA10g15590D)-chloroplastic/cytoplasmic and one gene was encoding trans-cinnamate 4-monooxygenase (BnaA03g14000D) enzymes. The three mentioned genes were responsible for the biosynthesis of phenylalanine or phenylpyruvate and trans-4-hydroxycinnamate, respectively.

Overall, most of the genes involved in the pathways leading to the accumulation of phenylacetamide (meta_741), succinate (meta_211), 2-Hydroxyphenylacetic acid (meta_266), N-Acetyl-L-phenylalanine (meta_390), and 3-Phenylpropanoic acid (meta_75), while salicylic acid (meta_92) was downregulated. A few genes were upregulated based on the three comparisons (Figure 10A), suggesting the DEGs were expressed at a higher level in CT-C18. However, other final or intermediate products of phenylalanine metabolism exhibited no difference between the two varieties except for the three DAMs. These findings suggested that rapeseed responded to CS by normalizing amino acid accumulation through phenylalanine metabolism. Nonetheless, having higher contents of these DAMs, mainly in CT-C18, may be associated with CS tolerance in rapeseed.

### Validation of Transcriptome and Metabolome Data

Six DEGs were randomly selected (involved in the starch and sucrose and phenylalanine metabolisms) to validate the RNA-seq data with qRT-PCR. As demonstrated in Supplementary Figure 7, all the selected genes display similar expression patterns to those generated from RNA-seq data, thus indicating the reliability of the RNA-seq data. Likewise, five DAMs were also validated by the targeted metabolite detection method, and the L-Tyrosine contents were significantly increased at 1 and 7 days of stress in both varieties. Similarly, L-Kynurenine contents were significantly increased at 7 days rather than 0 and 1 day. On the other hand, the succinic acid contents were reduced at 7 days in both varieties, while the N-Acetyl-L-Phe content was notably increased at 7 days in the CT-C18 variety than CS-C6. Similarly, the salicylic acid content was increased at 7 days in the CT-C18 variety compared to 0 and 1 day of CS treatments (Supplementary Figure 8). The noticed accumulation trends were consistent for selected metabolites, indicating the reliability of the metabolome data.

### Functional Validation of Candidate Genes

**Figure 11:**
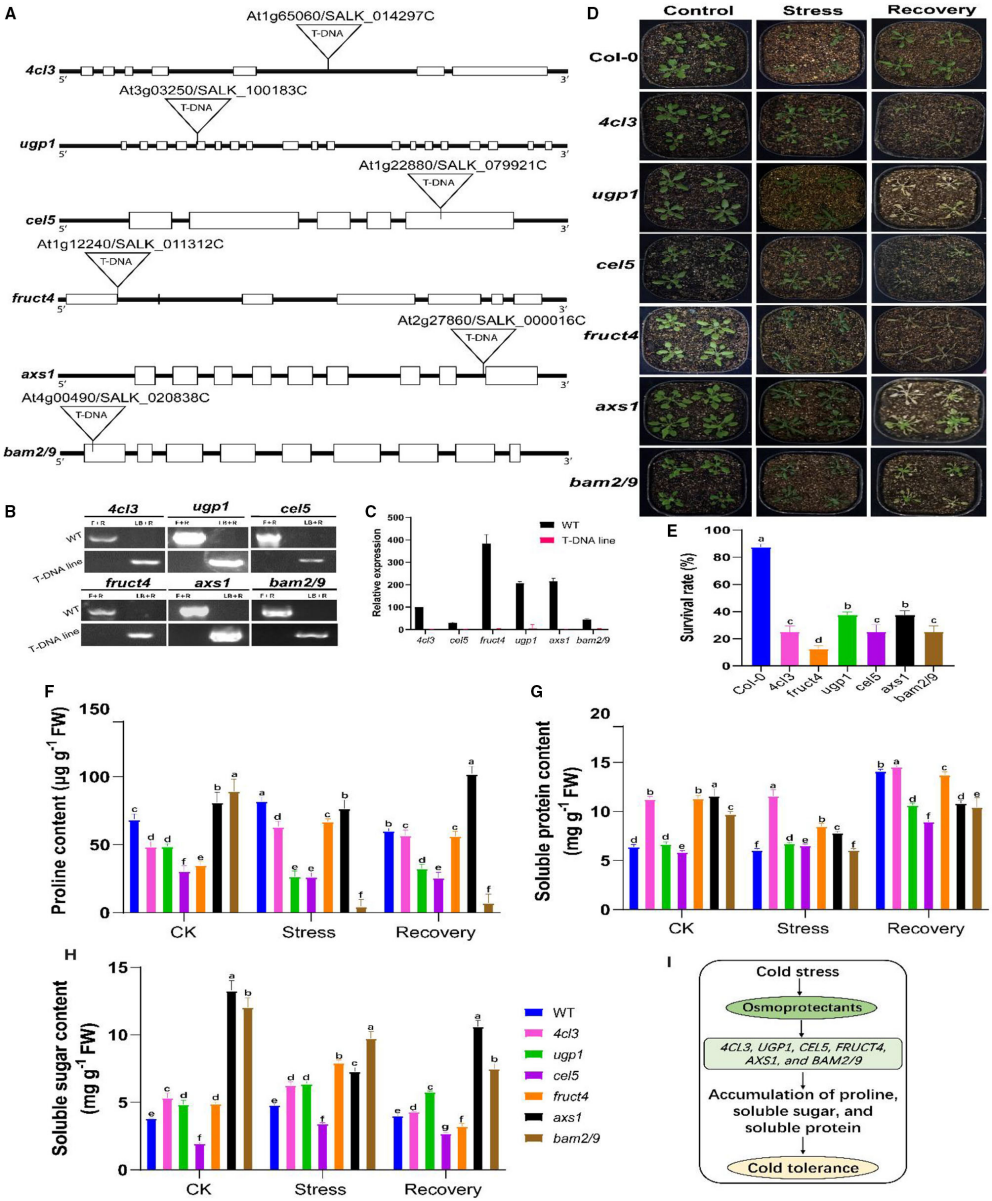
Functional validation of selected candidate genes using *Arabidopsis* mutants. **(A)** Schematic diagram of the gene structure of *4cl3* (4-coumarate coenzyme A ligase 3), *ugp1* (UDP-glucose pyrophosphorylase 1), *cel5* (CELLULASE 5), *fruct4* (beta-fructosidase 4), *axs1* (UDP-D-xylose synthase 1/UDP-D-apiose), and *bam2/9* (beta-amylase 2/9). Exons and introns are indicated in black boxes and black lines, respectively. **(B)** PCR-based genotyping of these mutants. **(C)** Relative expression of mutant genes in wild-type (WT) and mutant plants. **(D)** Phenotypic evaluation before stress [control (CK) at 25°C], 8 h of freezing stress (−5°C), and after 2 days of the recovery phase (recovery at 25°C). **(E)** Analysis of survival rates in the leaves of the above-mentioned mutants and WT plants during the freezing treatment. **(F–H)** Analysis of the osmotic substances (proline, soluble protein, and soluble sugar) in selected *Arabidopsis* mutants before stress (CK), after stress (−5°C for 8 h), and after 2 days of recovery at 25°C. **(I)** A working proposed model for the role of osmoprotectants in mutants for cold tolerance. Upon exposure to cold stress, the accumulation of osmotic substances helps improve cold tolerance. Error bars represent SD (*n* = 3) of the three biological replicates in **(D)** and the mean of the three technical replicates **(E–G)**. The statistical significance was determined *via* one- or two-way ANOVA and Dunnett's multiple comparisons test. Different lowercase letters on the error bars indicate significant differences at *p* ≤ 0.05.

For functional validation, some candidate genes were selected from the starch and sucrose metabolism and phenylalanine metabolism pathways. Previous studies showed that the use of *Arabidopsis* homologues T-DNA insertion mutants is a very suitable approach for functional validation, which has functional elements of targeted genes and can provide insights into the key role of a particular gene under a given environment (Jia et al., 2016; Melencion et al., 2017; Yu et al., 2020). Our selections of genes were based on the hypothesis that mutations (lack-of-function) of these candidate genes would affect CS tolerance levels in rapeseed. To date, the homologues of selected rapeseed genes, including *Bn4CL3* (BnaC05g30950D), *BnCEL5* (BnaA02g15500D), *BnUGP1* (BnaCnng53900D), *BnFRUCT4* (BnaC08g41390D), *BnAXS1* (BnaC08g43300D), and *BnBAM2/9* (BnaCnng02280D/BnaA09g51890D) have not been characterized. The sequence similarity between respective rapeseed and *Arabidopsis* lines were ranged from 69 to 96% (Supplementary Figure 9). Hence, T-DNA insertion mutants for the *Arabidopsis* homologues including *4cl3* (SALK 014297C), *cel5* (SALK 079921C), *ugp1* (SALK 100183C), *fruct4* (SALK 011312C), *axs1* (SALK 000016C), and *bam2/9* (SALK 020838C) were functionally validated under freezing stress conditions (Figure 11). The phenotypic evaluation results showed that the leaves of the mentioned mutants concurrently suffered severe freezing injuries and existed with low survival rates compared to the control plants after the freezing treatment, whereas the mutants of the homologues of *4cl3, cel5, fruct4, ugp1, axs1*, and *bam2/9* were sensitive to freezing stress (Figures 11E,F). These outcomes support our hypothesis that the lack-of-function of these genes could cause sensitivity to CS in rapeseed. On the other hand, these overexpression of these genes (in future works) could significantly improve CS tolerance in rapeseed plants by regulating sugar and amino acid contents in a particular metabolism.

According to our hypothesis, to get further mechanistic insights into the mechanisms behind CS tolerance, we evaluated osmoprotectant substances, including proline (Pro), soluble sugar (SS), and soluble protein (SP), and their contents in *Arabidopsis* mutants (Figures 11F–H). Results showed that all the mutants greatly differed in Pro, SS, and SP contents before and after stress and after recovery at 25°C. For example, *4cl3* showed a higher accumulation of Pro, SP, and SS than CK (Figures 11F–H), while, in other mutants, it was noticed that the contents of osmoprotectants were increased after stress, and, in some cases, a slight reduction was observed at the recovery stage compared to CK. However, this was not the case in all mutants. For instance, *cel5* did not significantly differ in the osmoprotectant content; whereas, in *bam2/9*, the SS, SP, and Pro, contents were decreased after stress treatment compared to CK. These results agreed with our hypothesis that CS leads to the accumulation of osmoprotectants, which helps plants minimize the adverse effect of CS (Figure 11I). Overall, it can be concluded that these candidate genes were expected to play a significant role in CS response and tolerance in rapeseed. In the future, genetic analysis can be carried out in rapeseed to understand the key role of these genes. Hence, these results confirmed the influence of conjoint analysis in revealing the genetic mechanisms of CS response and presented a set of candidate genes for improving CS tolerance in rapeseed.

## Discussion

Cold stress is one of the crucial environmental factors affecting the growth and development of plant species (Hu et al., 2017; He et al., 2021). Plants have evolved governing mechanisms that can respond to CS, including alterations in gene expression and metabolic profiles (Raza, 2020a; Mehmood et al., 2021; Raza et al., 2021b). Different genotypes have revealed different abilities to sustain growth and production. Therefore, it is important to use high-throughput omics approaches to identify CS-responsive genes or metabolites (Raza et al., 2021a,b). In the current study, an integrated analysis of transcriptomes and metabolomes during CS treatment was carried out in the CT-C18 and CS-C6 varieties, which allowed us to gain insights into the key metabolites, genes, and metabolic pathways involved in CS adaptation and tolerance in rapeseed.

### General Metabolome Analysis Revealed Stress Responses and Tolerance Mechanisms in Rapeseed

The plant kingdom comprises ~200,000 metabolites, majority of which are still unknown, reflecting their complicated roles in the natural life of plants. Metabolites are the final products of metabolism, playing substantial physiological and biochemical roles in several plant species under various environmental stresses (Foito and Stewart, 2018; Raza, 2020b). It has been reported that different sugars and amino acids such as trehalose, glucose, fructose, inositol, galactinol, raffinose, sucrose, putrescine, ascorbate, phenylalanine, and alanine would accumulate after CS in tobacco (Jin et al., 2017). However, the variation of metabolites in response to CS in rapeseed was yet to be reported. Thus, the current research also confirmed the fluctuation in the levels of the different metabolites in rapeseed varieties during CS treatment and the significant difference of DAMs belonging to carbohydrates, sugars, and amino acids between the two rapeseed varieties (Table 1). However, a huge number of metabolites that respond to multiple abiotic stresses, including CS in rapeseed and other major crop plants is unknown, unannotated, and yet to be reported.

For example, the adenosine 3′,5′-cyclic phosphate (cAMP; meta_1043) works as a secondary messenger upon binding activities protein kinase A (PKA), allowing the phosphorylation of protein substrates (Alqurashi et al., 2016). According to a previous report, cAMP shortage harmfully disturbs cell proliferation (Sabetta et al., 2016). In *Arabidopsis*, a cAMP-based proteome revealed the vital role of cAMP under salinity and CS conditions, suggesting that cAMP is crucial to induce multifaceted variations in cellular energy homeostasis (Alqurashi et al., 2016). In our study, the increased accumulation of cAMP was detected in C6-0 vs. C18-0 and C6-7 vs. C18-7 (Table 1, G2). Likewise, acetylcarnitine, alpha-D-glucose, and deoxyadenosine had a higher expression in short-term stress responses (Table 1, G3). The high quantities of cAMP and acetylcarnitine have also been reported by Djami-Tchatchou et al. (2019) in chili pepper inoculated with *Pectobacterium carotovorum*. Some ammonium molecules such as acetylcarnitine can be abundant in some soils and are thus taken up as intact compounds by plants. These ammonium compounds play a vital role in stress responses and rely on nitrogen cycling and plant nutrition (Warren, 2013). The detection of acetylcarnitine is becoming increasingly common in plants under normal and stressful conditions, e.g., plant developmental processes (Nguyen et al., 2016) and recovery from salinity stress (Charrier et al., 2012). Further, another important metabolite, trehalose, is composed of two alpha-D-glucose molecules. These molecules play a key role in stress tolerance and different developmental processes by the hydrolysis of trehalose. Hence, Liu et al. (2017) found the upregulation of alpha-D-glucose in okra metabolome analysis during postharvest senescence, confirming the vital role of trehalose in senescence.

Senescence plays a significant role in mobilizing nutrients from the roots to other plant parts for proper growth and development. In this regard, sugar molecules act as the main signaling molecules and help the movement of nutrients to cope with the current stress environment (Sami et al., 2016). In metabolome analysis, the upregulation of deoxyadenosine has been noted in maize seedlings when exposed to salinity stress (Yue et al., 2020) and in Tibetan hulless barley under CS (Yang et al., 2020).

Cytidine 5′-diphosphocholine (CDP-choline) is a nucleotide consisting of choline, cytosine, ribose, and pyrophosphate molecules and was upregulated in C6-1 vs. C18-1 and C6-7 vs. C18-7 comparisons. Several metabolome studies found the upregulation of CDP-choline, for instance, in halophilic microalga under salinity stress (Jiang et al., 2019). In another study, Sawada et al. (2017) reported the higher expression pattern of CDP-choline in a single-grain-based metabolome profiling of *Arabidopsis* seeds. Zhang et al. (2020) suggested that choline-mediated lipid reprogramming could lead to a salinity tolerance mechanism in non-glycine betaine accumulating Kentucky bluegrass (*Poa pratensis*). An untargeted metabolome profiling between japonica and indica rice cultivars revealed the upregulation of gamma-tocotrienol (a type of vitamin E) during network analysis (Hu et al., 2014). Recently, the metabolic profiling of DREB-overexpressing transgenic wheat seeds also indicated the upregulation of gamma-tocotrienol under different abiotic stress conditions (Niu et al., 2020). Tocotrienols arise in photosynthetic plants in variable quantities, and vegetable oils like sunflower, corn, safflower, and cottonseed deliver a valuable basis for these vitamin E forms (Ahsan et al., 2015).

Another metabolite, N-Acetyl-L-phenylalanine, had a higher expression in a long-term stress response (Table 1, G4). This metabolite has also been upregulated in *Atriplex halimus* metabolic profiling when exposed to salinity and drought stress (Alla et al., 2012). Moreover, the comparative metabolome and transcriptome analysis of different tissues of wheat plants documented the vital role of the upregulation of N-Acetyl-L-phenylalanine in stamen and pistil growth (Yu et al., 2019). These findings suggest that N-Acetyl-L-phenylalanine is another vital metabolite responsible for stress tolerance and several plant developmental stages.

The organic compounds Tyr-Asp were upregulated in a long-term stress response (Table 1, G4). Several metabolome-based investigations found the higher expression of this compound under different stress conditions. For instance, Sun et al. (2015) suggested that the upregulation of Tyr-Asp plays an important role in plant stress physiology and alters the plant metabolome profiling in wheat plants under drought and salt stress. In addition, squalene was down-regulated throughout the stress at all time points (Table 1, G1). A similar trend was also reported by Lu et al. (2016) under CS in *Nitrosomonas europaea*, as ammonia uptake patterns were altered.

In this study, trehalose, L-Kynurenine, gamma-tocotrienol, phenyllactic acid, and L-Gulonic gamma-lactone had increased contents throughout the stress in both varieties (Table 1, G1), suggesting the vital role of these DAMs in CS tolerance and adaptation. According to previous investigations, L-kynurenine has been characterized for auxin biosynthesis *via* the TAR/YUC pathways in plants (Brumos et al., 2018; Wang et al., 2018b). Under ammonium stress, the exogenous application of L-kynurenine inhibits root growth in rice, increases sensitivity to ammonium, and helps rice plants improve auxin biosynthesis under stress conditions (Di et al., 2018). Notably, gamma-tocotrienol, phenyllactic acid, and L-Gulonic gamma-lactone are actively involved in a wide range of biotic stresses (He et al., 2017; Fan and Song, 2018; Tóth et al., 2018; Dao et al., 2019). In the current study, these DAMs were upregulated and accumulated throughout the stress in both sensitive and tolerant varieties. Among these DAMs, trehalose, a naturally occurring sugar present in numerous organisms of plants, and several studies have demonstrated the beneficial role of trehalose against several abiotic stresses such as cold tolerance in rice (Fu et al., 2020), chickpeas (Farooq et al., 2017), and tomatoes (Liu et al., 2020). The overexpression of the trehalose-encoding gene, trehalose 6-phosphate synthase 11 (*TaTPS11*), improves the freezing tolerance in *Arabidopsis* (Liu et al., 2019). Notwithstanding the insights provided by these investigations, some of the identified metabolites are yet to be studied under various abiotic stresses. Furthermore, resolving some bottlenecks such as mistakes in the identification and annotation tools would lead to the precise identification of a huge number of metabolites under adverse environmental stress conditions.

### Roles of Enriched Pathways Identified by the Combined Analysis

In this study, the enrichment analyses (KEGG, BP-GO, and MapMan) of DEGs correlated with DAMs were significantly associated with amino acid and carbohydrate metabolisms (Figure 4 and Supplementary Table 7). As previous studies showed, amino acid and carbohydrate metabolic pathways play significant roles in the salinity stress tolerance of tomatoes (Zhang et al., 2017). Several amino acid pathways have been observed after drought stresses in chickpea plants (Khan et al., 2019). Similar observations (tryptophan, phenylalanine, and histidine metabolisms) have also been detected in a combined metabolome and phenome analysis of maize under drought stress conditions (Witt et al., 2012). The three essential metabolisms, such as phenylalanine, tryptophan, and tyrosine, are well-thought-out in plant metabolisms (Galili and Höfgen, 2002). To date, many combined-omics studies have detected the vital role of these three metabolites and metabolic pathways, such as in *Pinus radiata* under high-temperature stress conditions (Escandón et al., 2018), in chickpea under drought stress conditions (Khan et al., 2019), in tobacco under CS conditions (Zhou et al., 2019), and in *Sargassum fusiforme* under heat stress conditions (Xie et al., 2019). Interestingly, the high accumulation of several carbohydrates provides energy to plants and helps them to cope with multiple abiotic stresses (Gupta and Kaur, 2005; Zhang et al., 2017). In *Salvadora persica*, a similar trend of galactose metabolism, starch and sucrose metabolism, and phenylalanine metabolism were observed after water deficit stress (Rangani et al., 2020). In rice, fructose and mannose metabolism and glutathione metabolism were significantly enriched after drought stress (Ma et al., 2016). Additionally, carbohydrate metabolism has been reported to be critical for heat and drought stress in soybeans (Das et al., 2017), for drought stress in poplar (Jia et al., 2020), and for salinity stress in sesame (Zhang Y. et al., 2019). These observations provide further evidence for the significant role of amino acid and carbohydrate metabolic pathways in response to various abiotic stresses.

The ABC transporters were one of the major enriched biological pathways detected during CS, mainly in CS-C6 (Supplementary Figure 3B), which was consistent with previous reports. In tall fescue, ABC transporters have been identified in response to nitric oxide-modulated cadmium stress tolerance by a combined transcriptome and metabolome analysis (Zhu et al., 2020). The ABA transporters and/or proteins are mainly involved in translocating several substances such as carbohydrates, ions, lipids, xenobiotics, antibiotics, drugs, heavy metals, etc., within the plants (Theodoulou, 2000; Rogers et al., 2001; Martinoia et al., 2002; Jungwirth and Kuchler, 2006). These findings suggest that ABC transporters play a significant role in translocation and help plants to cope with stressful environments. However, the excessive accumulation of substances may also affect plant growth and productivity and make the plant susceptible to stress conditions. Overall, it can be concluded that most of the identified pathways were reported under different abiotic stress conditions. Nevertheless, their significant role in CS tolerance in different crop plants needs more investigation.

### The Crucial Role of Starch and Sucrose Metabolism in Stress Tolerance

In starch and sucrose metabolism, several DEGs were correlated with the five DAMs (Figure 9). In the subsequent part, we discussed the vital role of some important genes. For instance, beta-glucosidase 16 (BnaAnng19210D) was upregulated and associated with five DAMs (Figure 9). Beta-glucosidases are largely present in the vacuole and play a vital role in plant carbohydrate metabolisms, including cell wall modification, defense, plant hormone signaling, and secondary metabolism (Cairns et al., 2015). It also works in the hydrolysis of disaccharides, i.e., cellulose hydrolysis, by translating cellobiose to glucose (Singhania et al., 2013). Consequently, the tolerant genotype seemed to be maintaining disaccharides under CS conditions. In rice, beta-glucosidase was upregulated when exposed to ABA, MeJA, submergence, and salinity stress (Opassiri et al., 2007). The loss of beta-glucosidase in *Arabidopsis* (*AtBG1*) gives rise to the drought-sensitive phenotype, while overexpression of *AtBG1* led to improved drought tolerance in *Arabidopsis* (Lee et al., 2006).

The expression level of beta-amylase (BnaC02g12830D) was upregulated and correlated with four DAMs in both varieties (Figure 9). Glucans produced from starch pellets are hydrolyzed through beta-amylase to maltose (Smith et al., 2005), and starch is an ample storage carbohydrate formed in plants (Feike et al., 2016). In *Arabidopsis*, beta-amylases were upregulated under drought, salinity, and cold stresses (Seki et al., 2002). Furthermore, higher beta-amylase content was crucial for the persistence of rice seedlings throughout the premature growth phase and following seedling growth with exposure to flooding stress (Ella et al., 2010). In another study, Wang X. et al. (2017) reported that beta-amylase and beta-glucosidase activities were significantly increased by modifying carbohydrate metabolism. These discoveries advise that beta-amylase is elaborated in starch degradation in rapeseed exposed to CS. This enzyme might normalize carbohydrate utilization to upsurge energy facilities in the rapeseed seedlings. Moreover, the beta-fructofuranosidase gene (BnaA01g37220D/BnaC08g41390D) was upregulated and associated with five DAMs found in starch and sucrose metabolism (Figure 9). In Bambara groundnut landraces, Khan et al. (2017) also found the upregulation of the beta-fructofuranosidase gene in two genotypes grown under water-limited and water-sufficient conditions. Notably, beta-fructofuranosidase hydrolyzes the sucrose to produce additional glucose, thereafter playing an important role in osmoprotection and energy production in plants under stressful environments (Khan et al., 2017). The UTP-glucose-1-phosphate uridylyltransferase (BnaCnng53900D) showed higher expression and correlated with four DAMs in both varieties (Figure 9). As an important enzyme of carbohydrate metabolism and cell wall biosynthesis, it can catalyze the adjustable reaction among glucose-1-phosphate and UDP-glucose (Munoz-Bertomeu et al., 2010). As a glycosyl giver in cells, UDP-glucose can be elaborated in the glycosylation of several compounds in plant cells (Munoz-Bertomeu et al., 2010). In a combined root proteomic and metabolic study, the upregulation of UTP-glucose-1-phosphate uridylyltransferase was strongly linked to the drought stress responses in grapevine tolerant rootstocks (Prinsi et al., 2018). Under CS, Wang T. et al. (2017) observed that this enzyme plays a vital role in developing cold tolerance in *Anabasis aphylla* seedlings. Considering this, it can be concluded that the starch and sucrose metabolism was improved for contributing to CS tolerance in rapeseed.

### The Crucial Role of Phenylalanine Metabolism in Stress Tolerance

In phenylalanine metabolism, numerous DEGs were correlated with six DAMs (Figure 10A). Concisely, the biosynthesis of phenylalanine, tyrosine, and tryptophan begins with phenylalanine, which leads to the biosynthesis of other metabolites. Here, the 4-Hydroxyphenylpyruvate dioxygenase (HPPD)-encoding gene (BnaC08g44820D) was upregulated and correlated with five DAMs (Figure 10). The HPPD is an enzyme involved in photosynthesis regulation and catalyzes the conversion of 4-hydroxyphenylpyruvic acid (HPPA) into homogentisic acid (HGA) (Ndikuryayo et al., 2017). The HPPD enzyme-encoding genes play an essential role in pyomelanin synthesis (pigment produced by microbes) (Ahmad et al., 2016). Recently, the mutation analysis of pyomelanin production revealed the protective role of the pigment against oxidative stress tolerance in *Ralstonia solanacearum* (Ahmad et al., 2016). According to Kohlhase et al. (2018), waterhemp populations were found to be resistant to HPPD-inhibitor herbicides. In the future, the isolation of such a pigment from several bacteria grown in harsh environmental conditions may serve as a useful molecule for abiotic stress tolerance in plants.

Furthermore, aspartate aminotransferase (AAT)-encoding genes (BnaA05g11710D, BnaC01g06460D, and BnaA10g15590D) were correlated with 2-phenylacetamide, N-Acetyl-L-phenylalanine, and succinate DAMs (Figure 10). It has been reported that AAT catalyzes a changeable reaction using coenzyme pyridoxal-5-phosphate (PLP), which subsequently creates aspartate and 2-oxoglutarate and vice versa (Toney, 2014). Recently, it was noticed that the upregulation of *AAT* (LOC105167001) in the salinity-tolerant soybean variety was stronger than that in the sensitive variety (Zhang Y. et al., 2019). Consistently, it also showed a significantly higher expression level in the CT-C18 variety. Moreover, one AAT-encoding gene (*AlaAT*) from *Medicago sativa* was overexpressed in rapeseed, and the transgenic rapeseed lines presented promising improvement in biomass under differently fertilized conditions (McAllister et al., 2016). Thus, even genes functioning in producing vital compounds were found to be downregulated in both varieties. However, members of these compounds exhibited higher expression levels in CT-C18 like AAT, which might positively impact the stress tolerance of rapeseed. The current study also detected the downregulation of two genes encoding AAT (chloroplastic/cytoplasmic) in CS-C6, indicating that absence or low expression may give rise to the stress-sensitive genotype.

Additionally, the trans-cinnamate 4-monooxygenase (BnaA03g14000D) gene was downregulated and correlated with five DAMs in both varieties (Figure 10). In plants, phenylalanine ammonia-lyase (PAL) catalyzes phenylalanine and tyrosine to produce cinnamic acid and *p*-cinnamic acid, respectively. Cinnamic acid can be catalyzed by trans-cinnamate 4-monooxygenase (C4H) to produce *p*-cinnamic acid (Tohge et al., 2017). Monooxygenase reactions are important in the biosynthesis of diverse metabolites, including fatty acids, phenylpropanoids, alkaloids, and terpenoids, in plants (Tohge et al., 2017). Recently, a *BnGC4H* gene was characterized from cultivated *Boehmeria nivea*, with results showing that it was strongly expressed in the xylem during the maturity stage, which indicated its crucial role in the developmental process. Thus, these discoveries advised that CS tolerance of rapeseed could be related to normalizing amino acid accumulation and/or a breakdown in phenylalanine metabolism.

## Conclusion

The present study performed a conjoint analysis based on transcriptomes and metabolomes to identify the key genes, metabolites, and metabolic pathways related to CS response in tolerant and sensitive rapeseed varieties. It provided a total of 3,368 DAMs, among which 626 have already been annotated. Combining the transcriptome data, a total of 2,845, 3,358, and 2,819 DEGs from the comparison of C6-0 vs. C18-0, C6-1 vs. C18-1, and C6-7 vs. C18-7 were detected, respectively. Further analysis of these DAMs and DEGs displayed that carbohydrate and amino acid metabolisms, specifically starch and sucrose metabolism and phenylalanine metabolism, might be involved in the complex response or regulation of CS tolerance in rapeseed. Thus, a functional validation based on the *Arabidopsis* T-DNA insertion mutants revealed that *Bn4CL3, BnCEL5, BnFRUCT4, BnUGP1, BnAXS1*, and *BnBAM2/9* could be considered as strong candidates in adapting to CS in rapeseed. Therefore, these candidate genes could be used for future rapeseed genetic improvement programs. The combined analysis expanded our understanding of the critical metabolic pathways that influence rapeseed adaption to CS stress. Lastly, the DAMs or DEGs identified here may be exploited for agronomic improvement under cold stress conditions.

## Data Availability Statement

The original contributions presented in the study are publicly available. This data can be found at: National Center for Biotechnology Information (NCBI) BioProject database under accession number PRJNA596550.

## Author Contributions

AR and YL conceived the idea and designed the experiment. AR performed the experiments, analyzed the data, and wrote the manuscript. WS, MAH, and SSM participated in the experiments and analysis. YC, YL, XZh, and XZo supervised, proofread, and edited the manuscript. All authors have read and approved the final version of the manuscript.

## Conflict of Interest

The authors declare that the research was conducted in the absence of any commercial or financial relationships that could be construed as a potential conflict of interest.

## Publisher's Note

All claims expressed in this article are solely those of the authors and do not necessarily represent those of their affiliated organizations, or those of the publisher, the editors and the reviewers. Any product that may be evaluated in this article, or claim that may be made by its manufacturer, is not guaranteed or endorsed by the publisher.
