# Supplementary material for: Integrated Analysis of Metabolome and Transcriptome Reveals Insights for Cold Tolerance in Rapeseed (Brassica napus L.)
Source: Front Plant Sci. 2021 Oct 8;12:721681. doi: 10.3389/fpls.2021.721681 (PMC8532563; doi:10.3389/fpls.2021.721681)
Supplement: Supplementary file 1 [file Data_Sheet_1.ZIP › Supplementary figures.docx]

**Supplementary Figures**


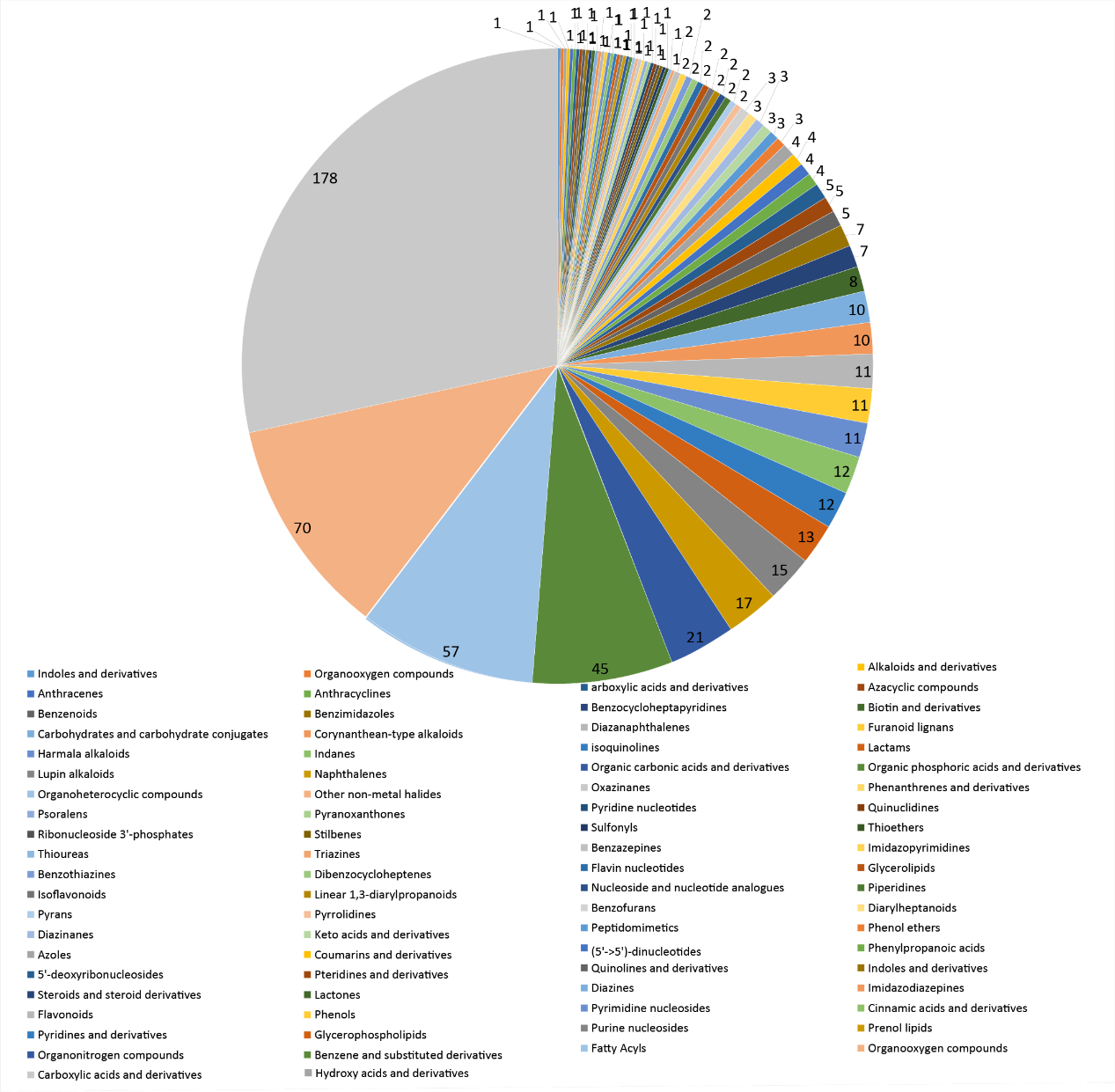


**Supplementary Figure S1** Classification of the 626 known identified metabolites in non-targeted metabolome analysis.


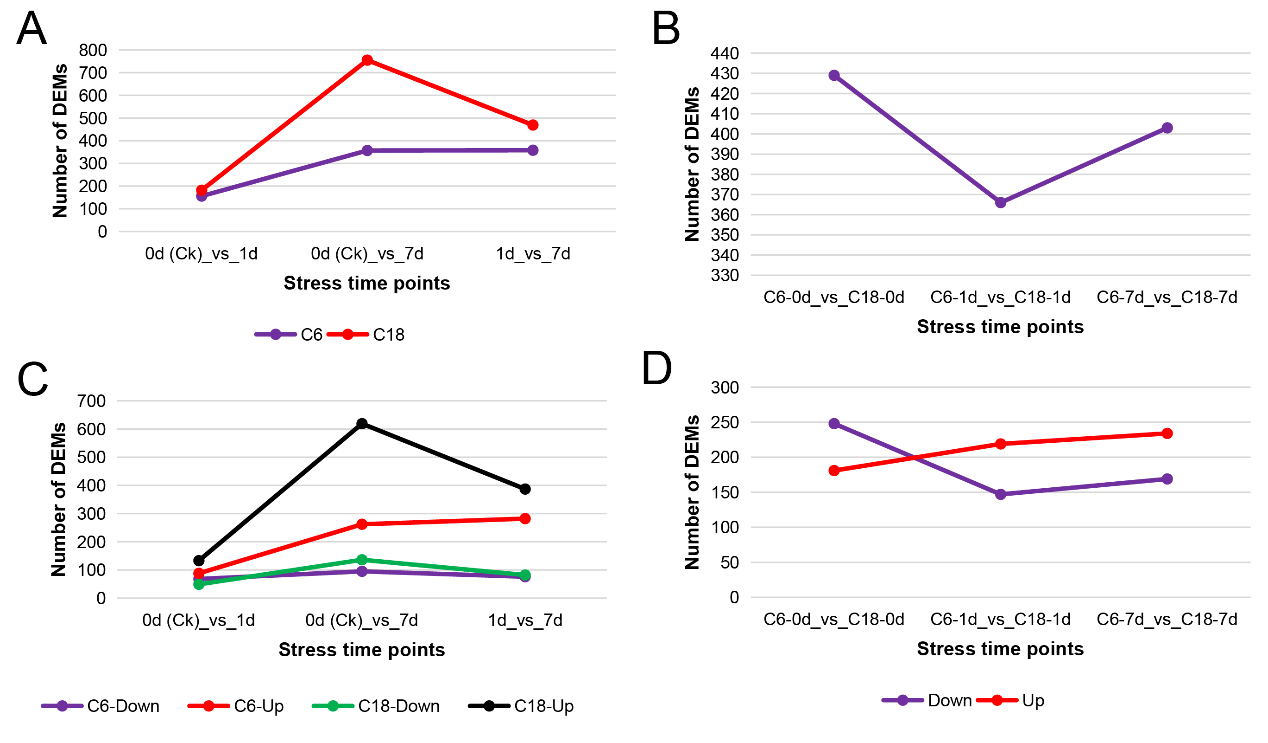


**Supplementary Figure S2** Alteration of metabolites in C6 and C18 under CS. (A) DAMs after 0 day, 1 day and 7 days after CS treatment; (B) DAMs between C6 and C18 at same time points; (C) Proportion of DAMs (up- and down-regulated) after 0 day, 1 day and 7 days after CS treatment; (D) Proportion of DAMs (up- and down-regulated) between C6 and C18 at same points.


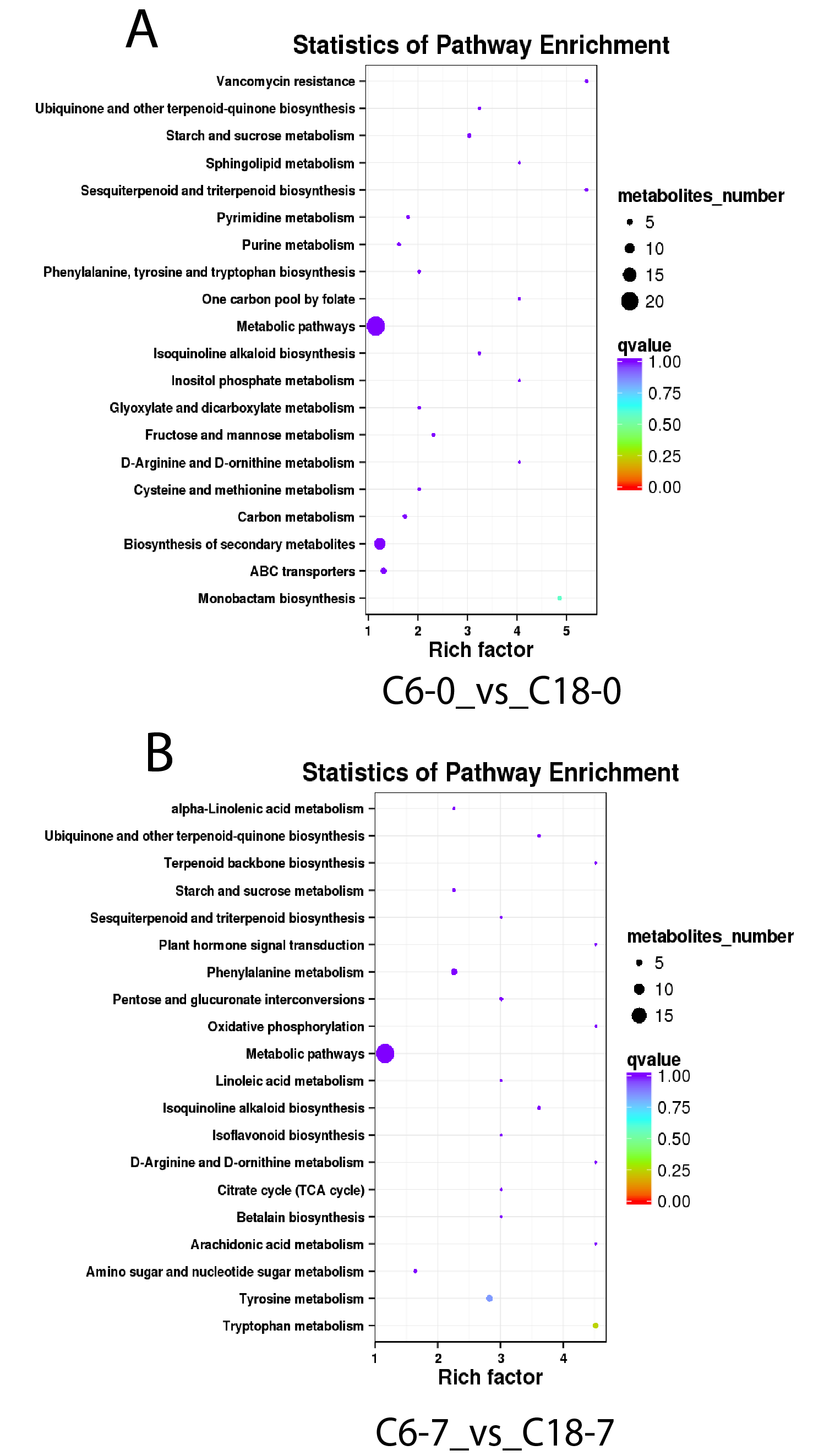


**Supplementary Figure S3** Scatter plots for KEGG pathway enrichment analysis of DAMs. **(A)** KEGG analysis of DAMs from C6-0_vs_C6-1. **(B)** KEGG analysis of DAMs from C6-0_vs_C6-7. Only highly enriched, most relevant KEGG pathways are shown in the plots (Q-value<0.05.) - log^10^ (Q-value) for enrichment factor.


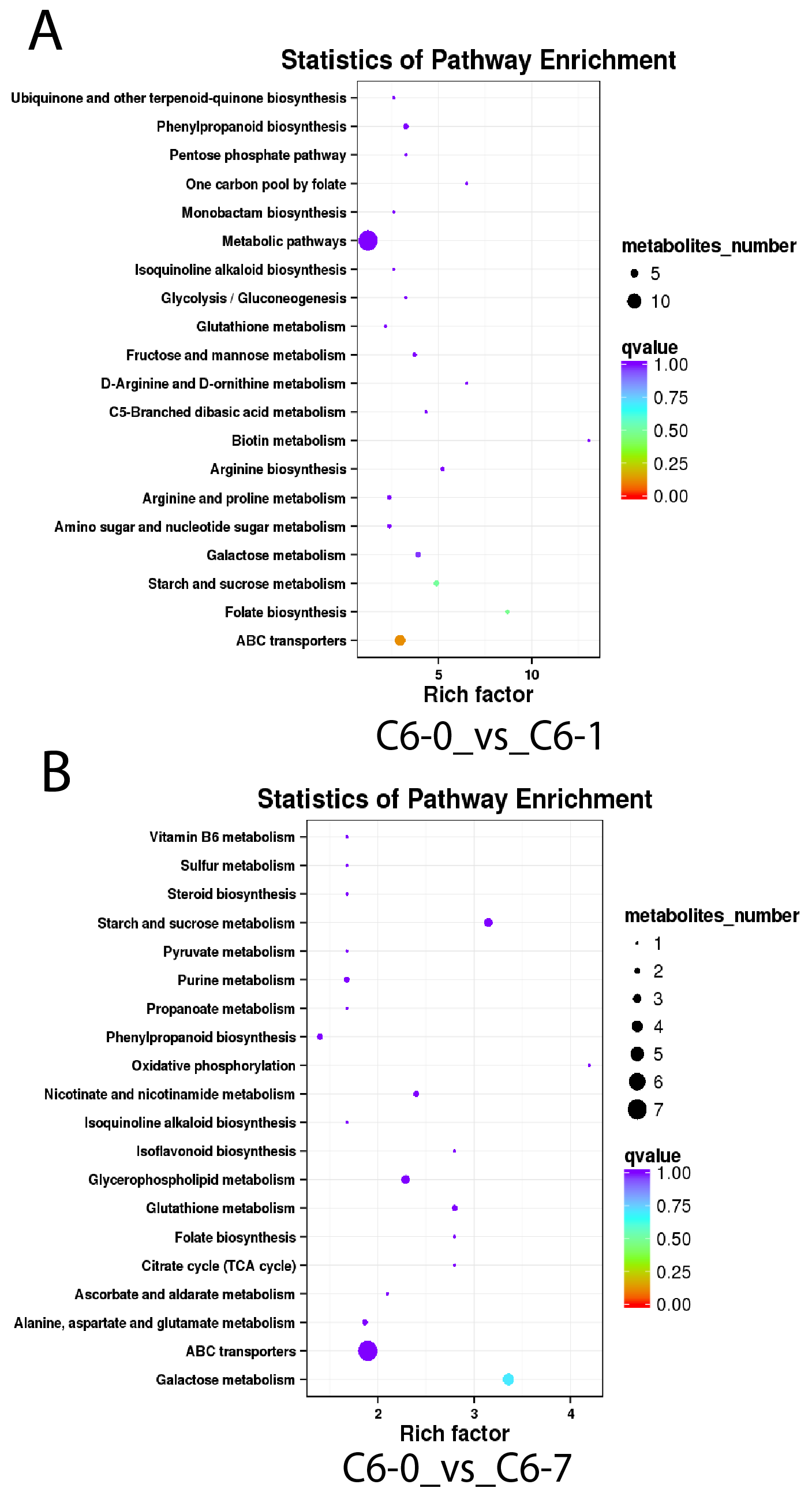


**Supplementary Figure S4** Scatter plots for KEGG pathway enrichment analysis of DAMs. **(A)** KEGG analysis of DAMs from C18-0_vs_C18-1. **(B)** KEGG analysis of DAMs from C18-0_vs_C18-7. Only highly enriched, most relevant KEGG pathways are shown in the plots (Q-value<0.05.) - log^10^ (Q-value) for enrichment factor.


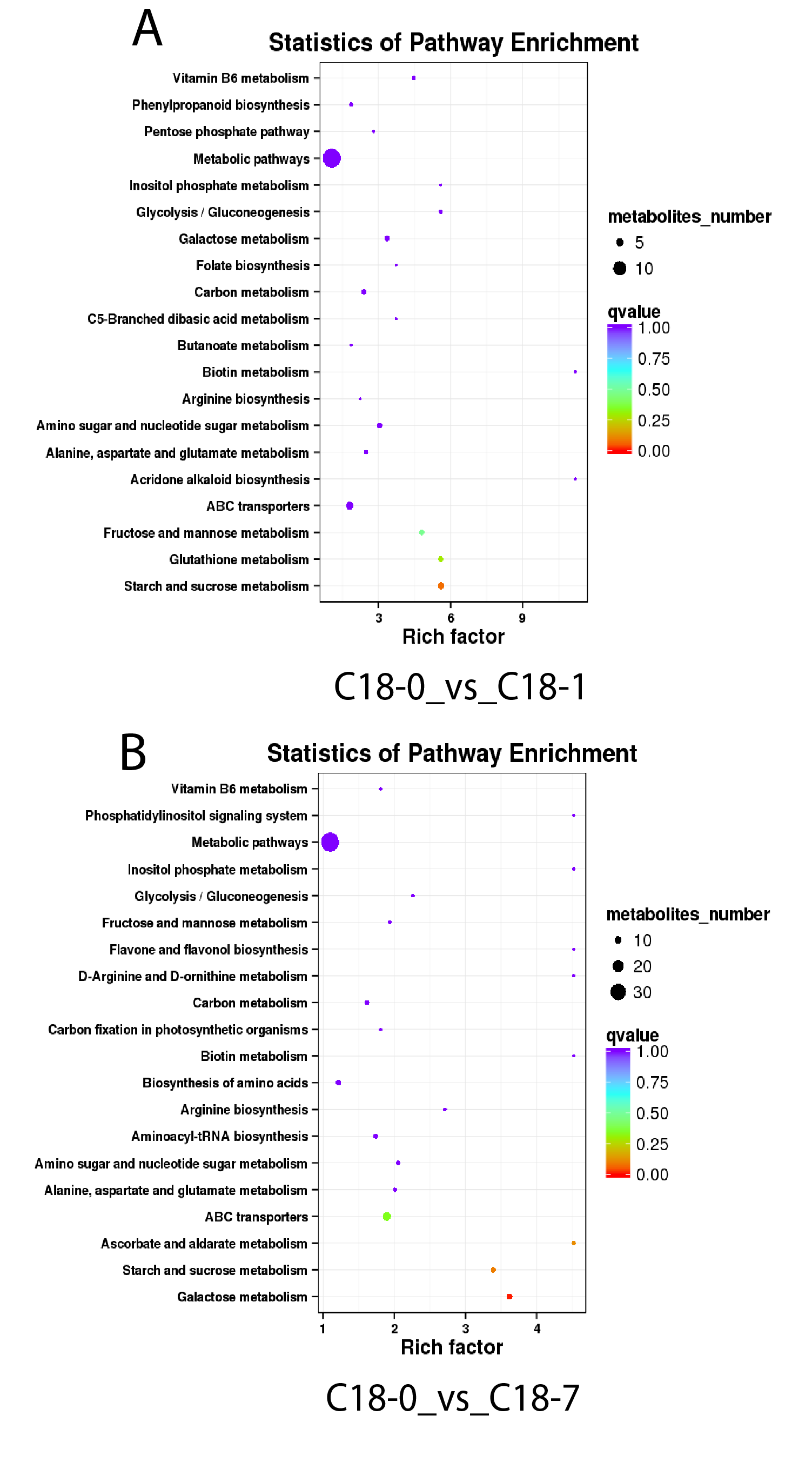
**Supplementary Figure S5** Scatter plots for KEGG pathway enrichment analysis of DAMs. **(A)** KEGG analysis of DAMs from C6-0_vs_C18-0. **(B)** KEGG analysis of DAMs from C6-7_vs_C18-7. Only highly enriched, most relevant KEGG pathways are shown in the plots (Q-value<0.05.) - log^10^ (Q-value) for enrichment factor.


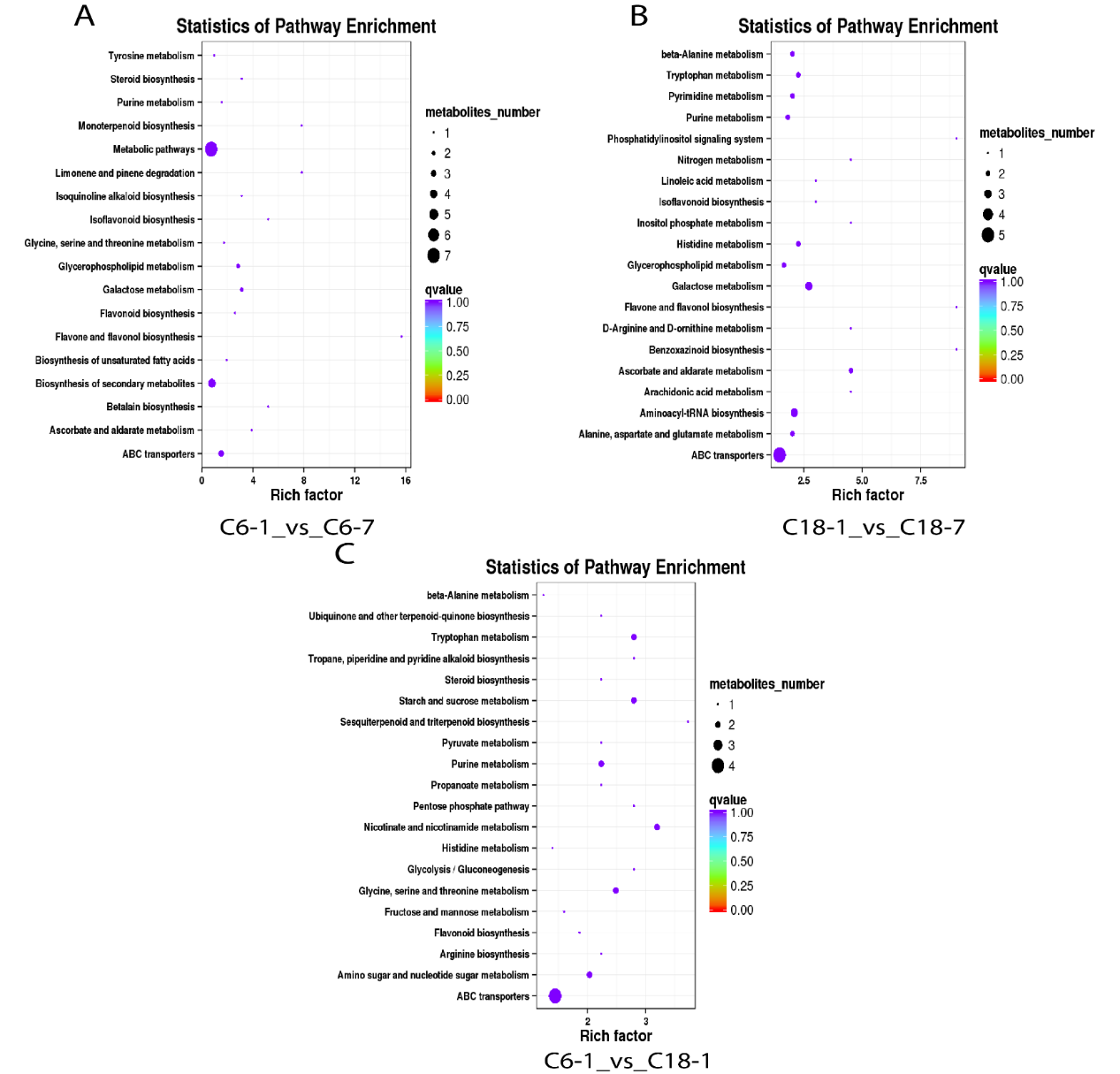


**Supplementary Figure S6** Scatter plots for KEGG pathway enrichment analysis of DAMs. (A) KEGG analysis of DAMs from C6-1_vs_C6-7, (B) KEGG analysis of DAMs from C18-1_vs_C18-7, (C) KEGG analysis of overlapped DAMs from C6 and C18 after 1 day of stress. Only highly enriched and relevant KEGG pathways are shown in the plots (Q-value<0.05.) - log^10^(Q-value) for enrichment factor.


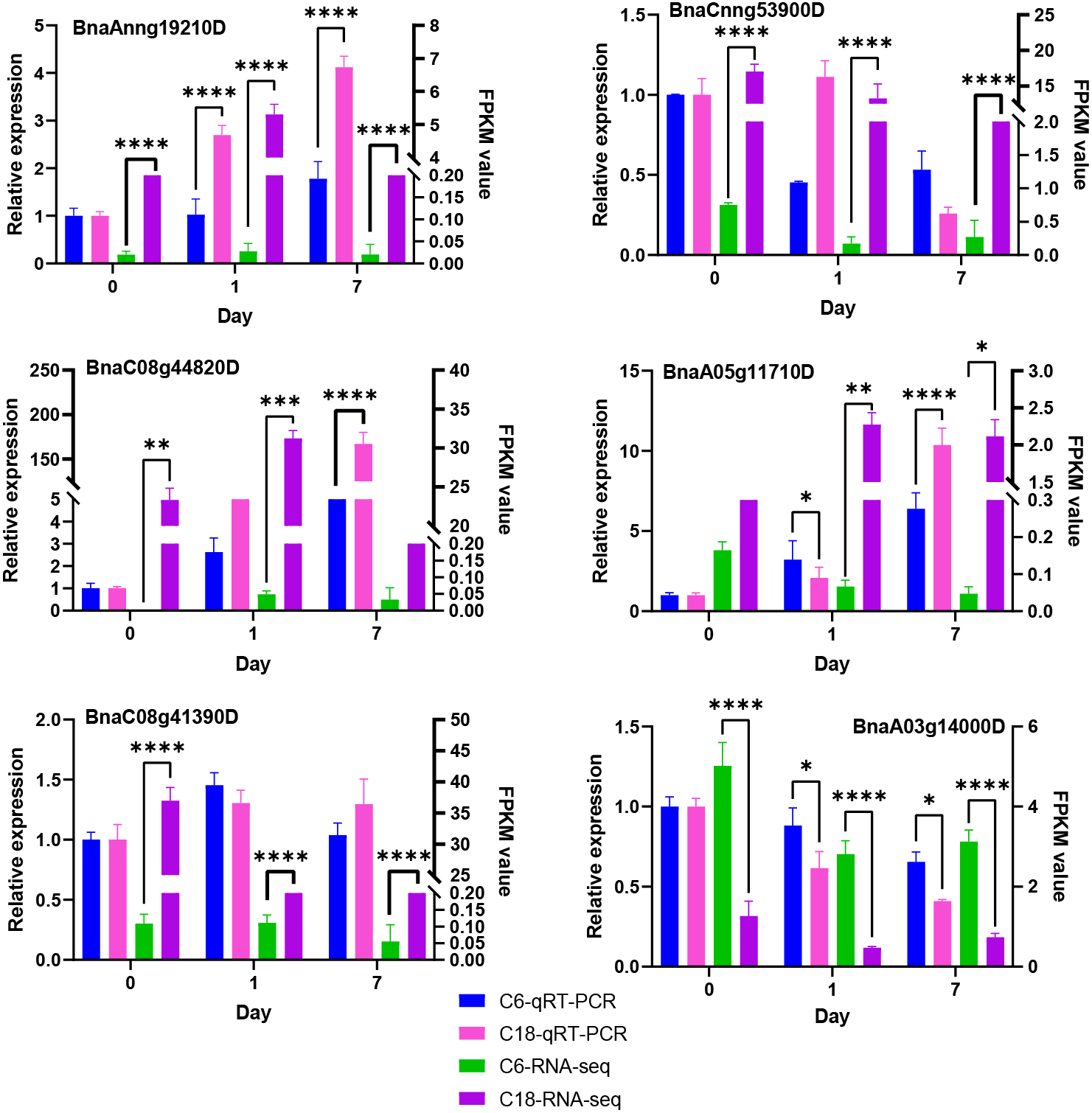


**Supplementary Figure S7** qRT-PCR validation of six randomly selected DEGs. Comparison of qRT-PCR with FPKM values of transcriptome data from C6 and C18 varieties. The relative expression level was presented on the left y-axis, and the FPKM values from the transcriptome data are revealed on the right y-axis. The expression level was stabilized using the averaged of the three technical replications. Each error bar denotes the mean of three biological replicates. The statistical significance was determined via Two-way ANOVA and Tuckey’s test with **** *p*< 0.0001, *** *p*≤ 0.001, ** *p*≤ 0.01, and * *p*≤ 0.05.

**
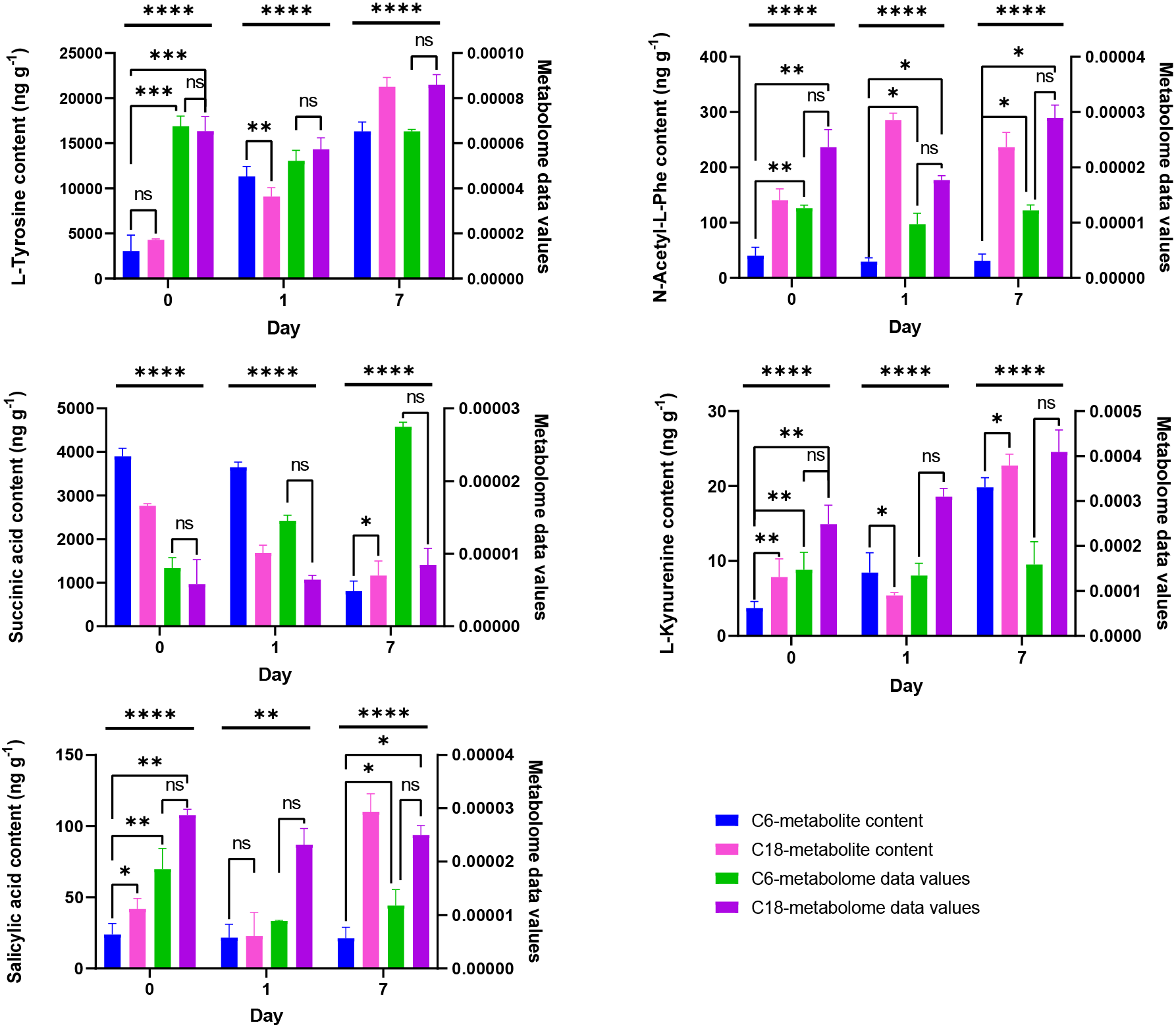
Supplementary Figure S8** Alteration of stress-induced metabolite contents in C6 and C18 genotypes under cold stress. Each error bar denotes the mean of three biological replicates. The statistical significance was determined via Two-way ANOVA and Tuckey’s test with **** *p*< 0.0001, *** *p*≤ 0.001, ** *p*≤ 0.01, and * *p*≤ 0.05.


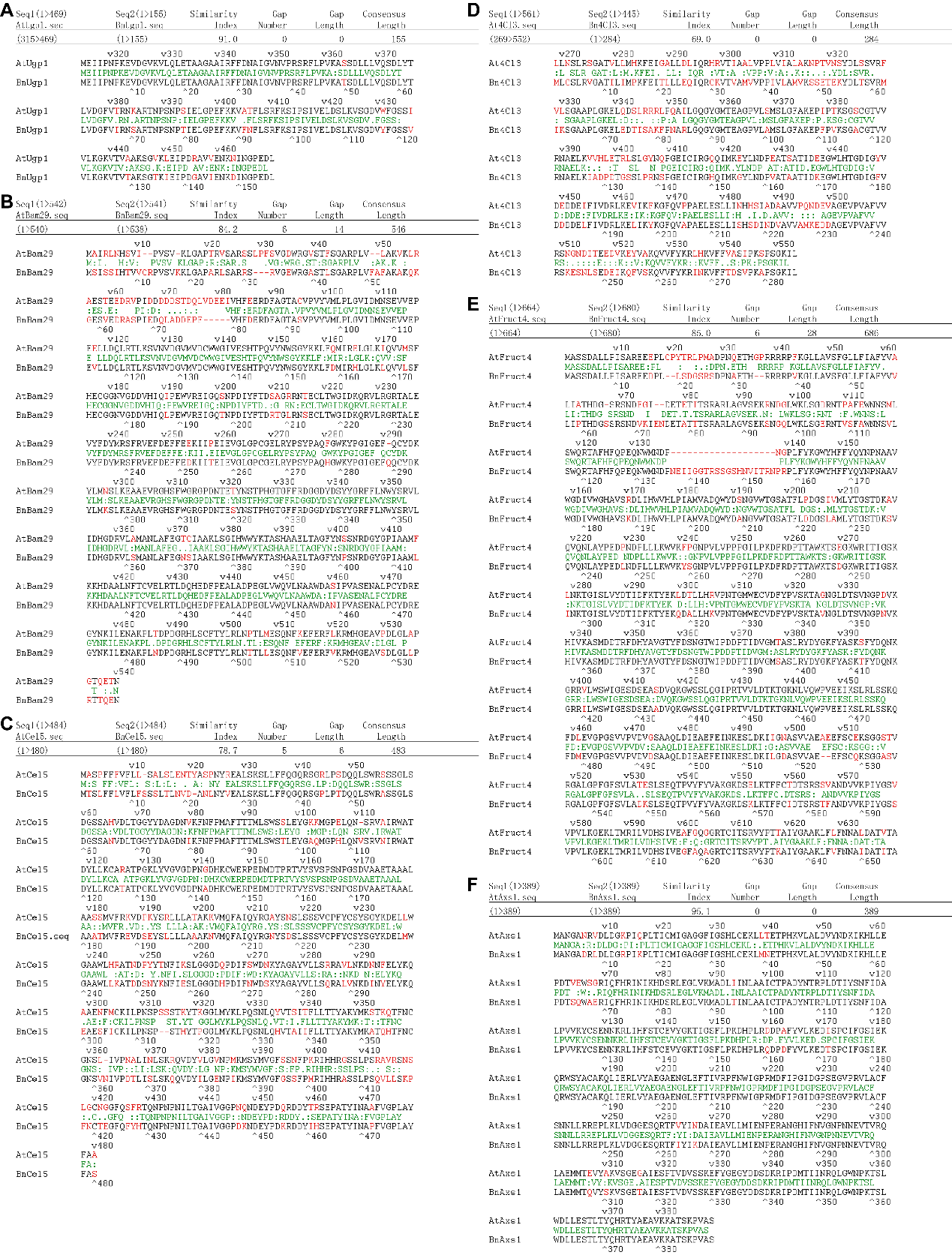


**Supplementary Figure S9** The sequence similarities between respective rapeseed and Arabidopsis T-DNA lines, including **(A)** ugp1, **(B)** bam2/9, **(C)** cel5, **(D)** 4cl3, **(E)** fruct4, and **(F)** axs1.
