# Supplementary material for: Integrated Analysis of Metabolome and Transcriptome Reveals Insights for Cold Tolerance in Rapeseed (Brassica napus L.)
Source: Front Plant Sci. 2021 Oct 8;12:721681. doi: 10.3389/fpls.2021.721681 (PMC8532563; doi:10.3389/fpls.2021.721681)
Supplement: Supplementary file 1 [file Data_Sheet_1.ZIP › Supplementary file S1.docx]

**2.10. Protocol used for the analysis of cold-induced alteration of some metabolites**

**1. Metabolite extraction**

An aliquot of each sample was precisely weighed and transferred to a 2 mL Eppendorf tube. After the addition of 1000 *μ*L of extraction solution (acetonitrile: methanol: water = 2:2:1), the samples were vortexed for 30 s, homogenized at 40 Hz for 4 min, and sonicated for 5 min in an ice-water bath. The homogenate and sonicate circle were repeated 3 times, and centrifugation at 12000 rpm and 4 °C for 15 min. The clear supernatant was transferred to an auto-sampler vial for UHPLC-MS/MS analysis. Another aliquot of the clear supernatant was further diluted 10 times for UHPLC-MS/MS analysis.

Stock solutions were individually prepared by dissolving or diluting each standard substance to give a final concentration of 1 mmol/L. A 100 μL aliquot of each of the stock solutions was transferred to a 10 mL flask to form a mixed working standard solution. A series of calibration standard solutions were then prepared by stepwise dilution of this mixed standard solution.

**2 Targeted metabolite detection via UHPLC-MRM-MS analysis**

The UHPLC separation was carried out using an Agilent 1290 Infinity II series UHPLC System (Agilent Technologies), equipped with an Agilent ZORBAX Eclipse Plus C18 (2.1 mm × 150 mm, 1.8 *μ*m, Agilent Technologies, CA, USA). Mobile phase A was 0.1% formic acid in the water, and mobile phase B was methanol. The elution gradients are shown in Table 1. The flow rate was 300 *μ*L/min. The column temperature was set at 35 °C. The auto-sampler temperature was set at 10 °C, and the injection volume was 1 *μ*L.

Table 1 The information of elution gradient used for UHPLC-MRM-MS analysis.

| **Time (min)** | **Solvent A** | **Solvent B** | **Flow (*μ*L/min)** |
| --- | --- | --- | --- |
| 0.0 | 95% | 5% | 300 |
| 1.0 | 95% | 5% | 300 |
| 4.0 | 70% | 10% | 300 |
| 6.0 | 5% | 95% | 300 |
| 8.0 | 5% | 95% | 300 |
| 9.2 | 95% | 5% | 300 |
| 11.0 | 95% | 5% | 300 |

An Agilent 6495 triple quadrupole mass spectrometer (Agilent Technologies), equipped with an AJS electrospray ionization (AJS-ESI) interface, was applied for assay development. Typical ion source parameters were: capillary voltage = +3000 V, Nozzle Voltage = +1500/ V, gas (N2) temperature = 250 ^o^C, gas (N2) flow = 11 L/min, sheath gas (N2) temperature = 400 oC, sheath gas flow = 12 L/min, nebulizer = 35 psi.

The MRM parameters for each of the targeted analytes were optimized, by injecting the standard solutions of the individual analytes directly, into the API source of the mass spectrometer. At least two MRM transitions (*i.e.*, the Q1/Q3 pairs) per analyte were obtained, and the two most sensitive transitions were used in the MRM scan mode to optimize the collision energy for each Q1/Q3 pair. Among the two MRM transitions per analyte, the Q1/Q3 pairs that showed the highest sensitivity and selectivity were used as the MRM transitions for quantitative monitoring. The additional transitions acted as a qualifier to verify the identity of the target analytes. Agilent MassHunter Work Station Software (B.10.00, Agilent Technologies) was employed for MRM data acquisition and processing.

**3 Calibration curves**

Calibration solutions were subjected to UPLC-MRM-MS/MS analysis using the methods described above. The least-squares method was used for the fittings. 1/x weighting was applied in the curve fitting since it provided the highest accuracy and correlation coefficient (R_2_). Minimum seven of fifteen concentration levels were included in the final calibration. The level was excluded from the calibration if S/N was close to or below 10, or the accuracy of the calibration was not within 80–120%.

**4 Limit of detection (LOD) and limit of quantitation (LOQ)**

The calibration standard solution was diluted stepwise, with a dilution factor of 2. These standard solutions were subjected to UHPLC-MRM-MS analysis. The signal-to-noise ratios were used to determine the limits of detection (LODs) and limits of quantitation (LLOQs). The LODs and LLOQs were defined as the analyte concentrations that led to peaks with signal-to-noise ratios (S/N) of 3 and 10, respectively, according to the US FDA guideline for bioanalytical method validation.

**5 Precision and accuracy**

The quantitation precision was measured as the relative standard deviation (RSD), determined by injecting analytical replicates of a QC sample. The accuracy of quantitation was measured as the analytical recovery of the QC sample determined. The percent recovery was calculated as [(mean observed concentration) / (spiked concentration)] × 100%.
