# Supplementary material for: Integrated Analysis of Metabolome and Transcriptome Reveals Insights for Cold Tolerance in Rapeseed (Brassica napus L.)
Source: Front Plant Sci. 2021 Oct 8;12:721681. doi: 10.3389/fpls.2021.721681 (PMC8532563; doi:10.3389/fpls.2021.721681)
Supplement: Supplementary file 1 [file Data_Sheet_1.ZIP › Supplementary Table S2.docx]

**Supplementary Table S2.1** List of primers used for qRT-PCR analysis of DEGs.

| S. No. | Gene ID | Forward primer | Reverse primer |
| --- | --- | --- | --- |
| 1 | BnaAnng19210D | GGTTTTAGACGGTTGACTAGGA | TATTATCAAAGACTACCGGCCC |
| 2 | BnaCnng53900D | GACCCGAGTTCAAGAAGGTTTT | AGTCACCTTGCCCTTGAGAAC |
| 3 | BnaC08g41390D | AGCGGAGTTCGAGATCAATAAA | GAAGTAAACCGGAGTTTGTTCC |
| 4 | BnaA05g11710D | AAACTTTGAAGCTTGCCTATGG | GGGATTAAAACGTGTCTGGAAG |
| 5 | BnaC08g44820D | GATGGTTCTGTTGCCGATAAAC | CCCTCAGCGTCCTGAATATATC |
| 6 | BnaA03g14000D | CTGGAGGAAGATGAGACGTATC | CACGGAACATGTTGTTGTACAT |

**Supplementary Table S2.2** List of primers used for PCR analysis of mutants.

| S. No. | SALK ID | Gene ID | Gene name | Forward primer | Reverse primer |
| --- | --- | --- | --- | --- | --- |
| 1 | SALK_014297C | At1g65060 | *4cl3* | ACGTGTCTCAAGGTGAACCAC | CTCAAGCTAATTTCGCATTGC |
| 2 | SALK_079921C | At1g22880 | *cel5* | CGTCTGTCCGTTCTACTGCTC | CCAACTTTAAAGTGAGTGGCG |
| 3 | SALK_011312C | At1g12240 | *fruct4* | ATTTTGCAATTGGTTGTGAGG | TACACGAGATTACCAATGGCC |
| 4 | SALK_100183C | At3g03250 | *ugp1* | ATCATTTCATGGATGCTTTGC | AACCAGACATCACCAGACACC |
| 5 | SALK_000016C | At2g27860 | *axs1* | GAGCTACGGTTTCACGTCAAG | TTCACCTAACAAAACCAACGC |
| 6 | SALK_020838C | At4g00490 | *bam2/9* | ATCCGTGACAATATCGCAAAG | TAGAAACACCACGCCAATCTC |

**Supplementary Table S2.3** List of primers used for qRT-PCR analysis of mutants.

| S. No. | Gene ID | Gene name | Forward primer | Reverse primer |
| --- | --- | --- | --- | --- |
| 1 | At1g65060 | *4cl3* | GACGTCATACTCTGCGTTTTAC | CCGCTATTGTCACTCTATGTCT |
| 2 | At1g22880 | *cel5* | CTACAAGGACGAGCTACTATGG | CTTTGTTTAGTACTGCTCGTCG |
| 3 | At1g12240 | *fruct4* | CAAGCAATTTGATCTCGAGGTC | GCGTTTCCGATGATTTTGTCTA |
| 4 | At3g03250 | *ugp1* | ATGAACAGTGGAAAGCTCGATA | ATTGTTAAGTCAACGATGGCAC |
| 5 | At2g27860 | *axs1* | TCCAGCTGATTACAATACACGT | GAAAAGTGAATGAGACGCTTGT |
| 6 | At4g00490 | *bam2/9* | TGGGGAATTGTTGAGTCACATA | TCTTAAGTCCAAGCTCACGAAT |
