# Supplementary material for: Integrated Analysis of Metabolome and Transcriptome Reveals Insights for Cold Tolerance in Rapeseed (Brassica napus L.)
Source: Front Plant Sci. 2021 Oct 8;12:721681. doi: 10.3389/fpls.2021.721681 (PMC8532563; doi:10.3389/fpls.2021.721681)
Supplement: Supplementary file 1 [file Data_Sheet_1.ZIP › Supplementary Table S7.doc]

**Supplementary Table 7.** KEGG pathway class, names and number of metabolites significantly accumulated in our study (hits).

| **S. no.** | **Class** | **Pathway name** | **Hits** |
| --- | --- | --- | --- |
| 1 | Amino acid metabolism | Histidine metabolism | 1 |
| 2 | Amino acid metabolism | Tryptophan metabolism | 1 |
| 3 | Amino acid metabolism | Tyrosine metabolism | 2 |
| 4 | Amino acid metabolism | Phenylalanine metabolism | 1 |
| 5 | Amino acid metabolism | Phenylalanine, tyrosine and tryptophan biosynthesis | 1 |
| 6 | Carbohydrate metabolism | Starch and sucrose metabolism | 1 |
| 7 | Carbohydrate metabolism | Galactose metabolism | 1 |
| 8 | Carbohydrate metabolism | Fructose and mannose metabolism | 1 |
| 9 | Carbohydrate metabolism | Glycolysis / Gluconeogenesis | 1 |
| 10 | Biosynthesis of other secondary metabolites | Monobactam biosynthesis | 1 |
| 11 | Biosynthesis of other secondary metabolites | Isoquinoline alkaloid biosynthesis | 1 |
| 12 | Membrane transport | ABC transporters | 2 |
| 13 | Lipid metabolism | Steroid biosynthesis | 1 |
| 14 | Metabolism of terpenoids and polyketides | Sesquiterpenoid and triterpenoid biosynthesis | 1 |
| 15 | Metabolism of cofactors and vitamins | Ubiquinone and other terpenoid-quinone biosynthesis | 2 |
| 16 | Nucleotide metabolism | Purine metabolism | 1 |
| 17 | Energy metabolism | Carbon metabolism | 1 |
| 18 |  | 2-Oxocarboxylic acid metabolism | 1 |
| 19 |  | Biosynthesis of amino acids | 1 |
